# Ontogenetic expression of thyroid hormone signaling genes: An *in vitro* and *in vivo* species comparison

**DOI:** 10.1371/journal.pone.0221230

**Published:** 2019-09-12

**Authors:** Kyla M. Walter, Katharina Dach, Keri Hayakawa, Susanne Giersiefer, Heike Heuer, Pamela J. Lein, Ellen Fritsche

**Affiliations:** 1 Department of Molecular Biosciences, University of California-Davis, School of Veterinary Medicine, Davis, CA, United States of America; 2 IUF–Leibniz Research Institute for Environmental Medicine, Dusseldorf, Germany; 3 Dept. Endocrinology, University Hospital Essen, Essen, Germany; University of Colorado Boulder, UNITED STATES

## Abstract

Thyroid hormone (TH) is essential for brain development. While disruption of TH signaling by environmental chemicals has been discussed as a mechanism of developmental neurotoxicity (DNT) for more than a decade, there remains a paucity of information linking specific TH disrupting chemicals to adverse neurodevelopmental outcomes. This data gap reflects, in part, the fact that the molecular machinery of TH signaling is complex and varies according to cell type and developmental time. Thus, establishing a baseline of the ontogenetic profile of expression of TH signaling molecules in relevant cell types is critical for developing *in vitro* and alternative systems-based models for screening TH disrupting chemicals for DNT. Here, we characterize the transcriptomic profile of molecules critical to TH signaling across three species–human, rat, and zebrafish–*in vitro* and *in vivo* across different stages of neurodevelopment. Our data indicate that while cultured human and rat neural progenitor cells, primary cultures of rat cortical cells, and larval zebrafish all express a fairly comprehensive transcriptome of TH signaling molecules, the spatiotemporal expression profiles as well as the responses to TH vary across species and developmental stages. The data presented here provides a roadmap for identifying appropriate *in vitro* and in simpler systems-based models for mechanistic studies and screening of chemicals that alter neurodevelopment via interference with TH action.

## Introduction

The potential for developmental exposure to environmental chemicals to cause neurodevelopmental defects by disrupting thyroid hormone (TH) activity has been under discussion for more than a decade [1–3]. This discussion is driven in large part by clinical evidence demonstrating that the thyroid hormones L-3,5,3',5'-Tetraiodothyronine (T4) and 3,3',5-triiodo-L-thyronine (T3) are essential for proper brain development. Children with severe prenatal TH deficiency exhibit symptoms including mental retardation, spasticity, deaf-mutism and hearing loss [4–6]. Less severe prenatal TH deficiency is associated with delayed mental and motor development, and lower IQ [7,8]. Conditions known to interfere with normal neurodevelopment by compromising TH action include iodine deficiency, maternal and fetal hypothyroidism or hypothyroxinemia, and mutations of the nuclear TH receptor (TR) alpha and beta as well as of monocarboxylate 8 transporter (MCT8) gene *SLC16A2* [9].

*In vitro* approaches have identified numerous chemicals with TH activity, but an outstanding question is whether hits in these screens are predictive of adverse neurodevelopmental outcomes. The majority of cell systems used to screen chemical compounds for effects on TH signaling are largely comprised of non-neural cells that overexpress molecules relevant for TH synthesis or direct TH genomic effects, i.e. the thyroid hormone receptor (TR) [10–13]. The former do not address potential effects at the level of the target cell, which may be a critical deficiency for developmental neurotoxicity (DNT) in light of experimental evidence demonstrating that chemical-induced decreases in circulating TH do not necessarily translate to decreased TH activity in the developing brain [14]. While the use of non-neural cells that overexpress distinct TR isoforms have the potential to detect actions of chemical compounds on cellular TR signaling, these models do not account for the known diversity of TH actions across different cell types that vary according to the role of TR and its binding partners including co-activators and co-repressors in cellular development and physiology [15]. Moreover, the presence and/or induction of deiodinases in target cells is a critical determinant of cellular TH action [16]. Thus, there is an urgent need to develop higher throughput models to screen chemical compounds for effects on TH signaling in developing brain cells. This will require cell systems that: *(i)* consist of cell types relevant for TH action in neurodevelopment, i.e. neurons and glia cells; *(ii)* are well-characterized with respect to TH actions and signaling pathways; *(iii)* represent defined developmental time windows with relation to TH function *in vivo*; and *(iv)* are representative of relevant TH biology.

**Fig 1:**
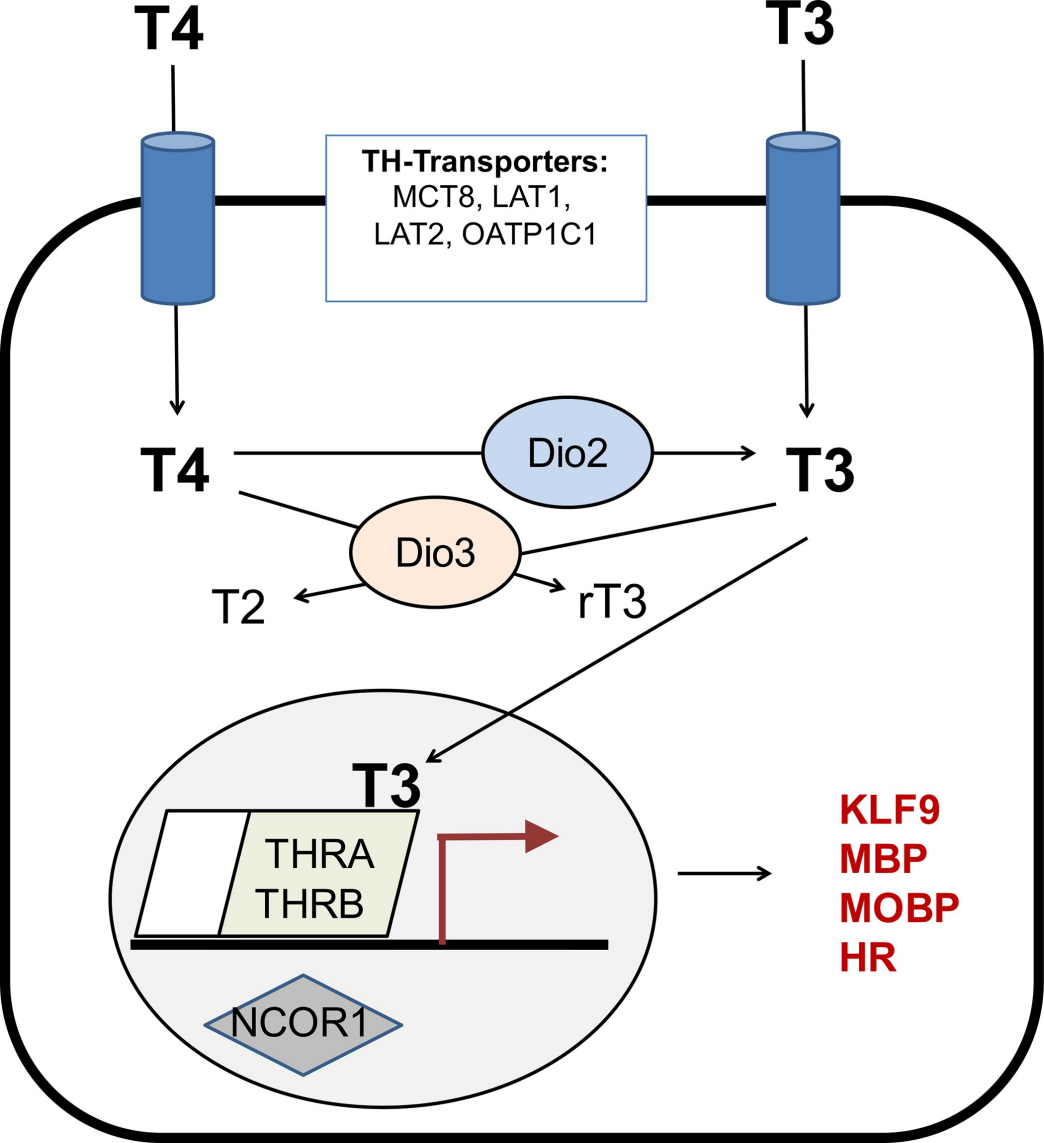
Schematic of the canonical TH signaling pathway. The canonical mechanism of TH action involves uptake of L-thyroxine (T4) or triiodothyronine (T3) into the cell by TH transporters, including the monocarboxylate transporter 8 (MCT8), L-type amino acid transporters (LAT) 1 and 2, and organic anion transporter protein (OATP) 1c1. Cellular deiodinase 2 (DIO2) converts T4 to T3, which has a higher binding affinity to the TH receptors (THR) α and β. DIO3 converts T4 to reverse T3 (rT3) and T3 to T2. The general model of TH regulation of gene transcription involves TH receptors (TRs; genes *THR*) that reside in the nucleus bound to DNA as homo- or possibly heterodimers with the retinoid X receptor (RXR) complexed with among others nuclear co-repressor 1 (NCOR1). Upon ligand binding, the TR-RXR complex releases NCOR1 and recruits activator proteins to initiate transcription of target genes, which include *DIO3*, *kruppel-like factor 9* (), *myelin basic protein* (*MBP*), *myelin-associated oligodendrocyte basic protein* (*MOBP*), and *hairless* (*HR*) [reviewed by 26]).

**Fig 2:**
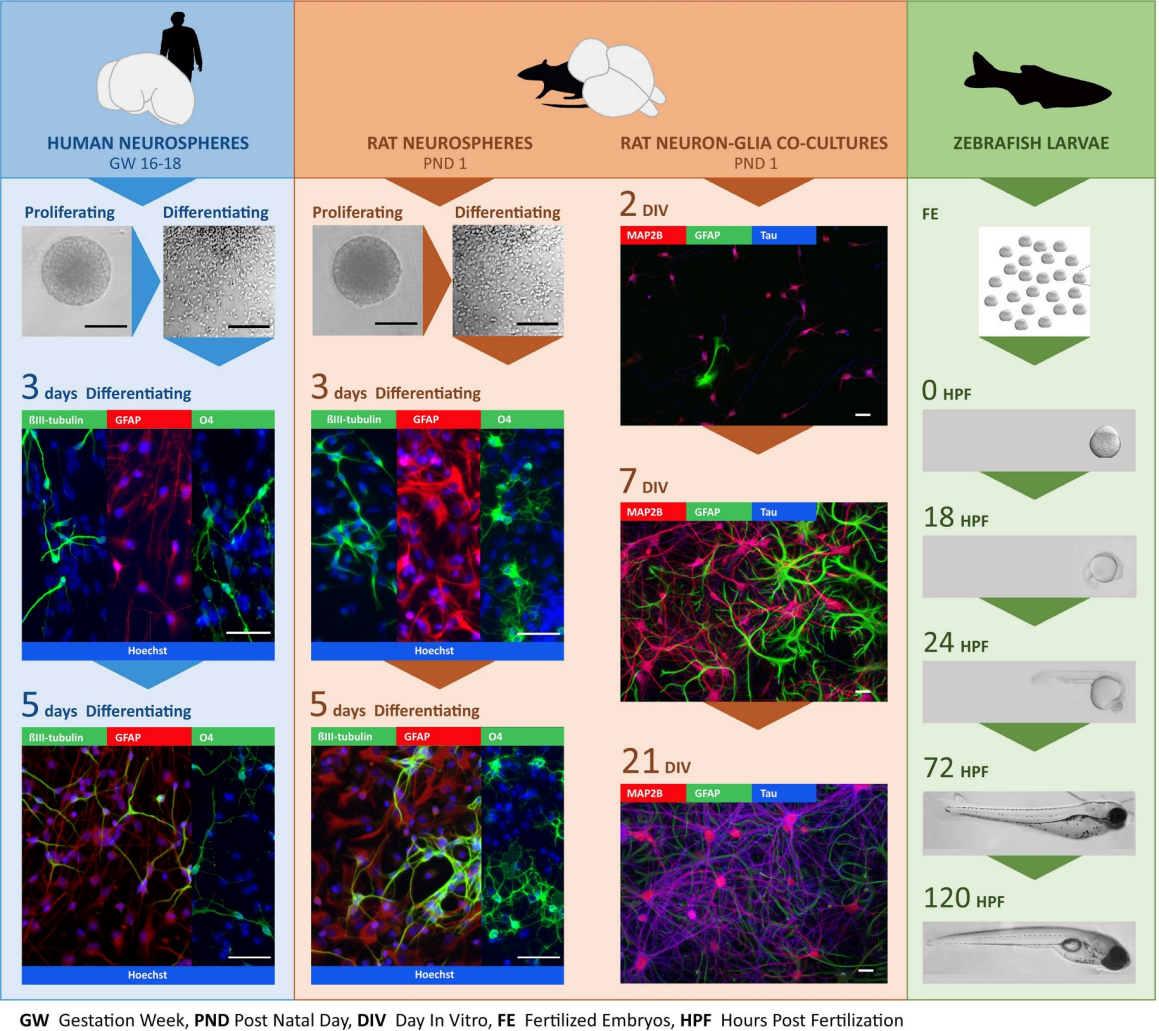
Overview of *in vitro* and *in vivo* models used for expression analyses of TH signaling-related genes. Human and rat neurospheres, primary rat cortical cell cultures and zebrafish larvae were used to study the expression of TH signaling components and TH-responsive genes at multiple developmental stages. In human and rat neurospheres, gene expression was evaluated in proliferating neurospheres and at days 3 and 5 of differentiation; in rat neuron-glia co-cultures at day *in vitro* (DIV) 2, 7 and 21, and in zebrafish larvae at 0, 18, 24, 72 and 120 hours post fertilization (hpf). The fluorescent photomicrographs illustrate the differentiation of the human and rat neurospheres and primary rat cortical cell cultures at these specific developmental stages; the transmission images, the morphology of the neurosphere cultures and zebrafish at different stages of development. The markers used for immunocytochemical analyses include βIII-tubulin (neurons), glial fibrillary acidic protein (GFAP; astrocytes), O4 (oligodendrocytes), microtubule-associated protein 2 (MAP2B; soma and dendrites of neurons), tau (axons of neurons), and Hoechst (cell nuclei). Scale bars are 200 μm (proliferating neuropheres), 150 μm (transmission images of differentiating neurospheres) or 50 μm (fluorescent photomicrographs).

Developing models to screen chemicals for their ability to directly interfere with TH action in the developing brain has been challenging, in part because the expression profile of key molecules involved in regulating TH activity in target cells (Fig 1) across different periods of brain development in different species is largely unknown. Therefore, the goal of this study was to address this data gap by comparing the transcriptomic profile of key TH signaling molecules, identified in Table 1, in various rat *versus* human *in vitro* models, as well as zebrafish larvae models. Because TH effects differ across developmental stages [17,18], and different *in vitro* models recapitulate different neurodevelopmental processes, we employed multiple *in vitro* model systems that captured differing periods of neurodevelopment. Fig 2 illustrates that the different models appear phenotypically as expected, i.e. containing neural cells of the neuronal and glial lineage at different maturation stages. One model we used was human neural progenitor cells (hNPC) derived from gestational week (GW) 16–18 brain tissue. In human fetal brains, TR transcripts are measurable as early as GW 10, and their concentration increases 10-fold until GW 16–18, which represents a 500-fold absolute gain in TR expression due to the increase in brain weight during that period [19]. The timing of brain development differs between species [20], but using an on-line tool for matching neurodevelopmental stages across species (www.translatingtime.org), we determined that human GW 16–18 corresponds approximately to postnatal day (PND) 0–1 in the rat. In the developing rat brain, *in vivo* T3 binding to TR is detectable at embryonic day E13.5–14, and reaches a maximum on PND 6 [21]. As previously described [22], these data regarding the ontogenesis of the TH signaling molecules in the human and rat, and the timing of analogous neurodevelopmental processes between these two species, provided the rationale for our decision to compare mRNA levels of critical TH signaling molecules in developing human and rat NPC from GW 16–18 and PND 1, respectively. NPC cultures capture early stages of neurodevelopment, such as neurogenesis, neuronal and glial cell differentiation and neural cell migration (Fig 2). To examine the expression of TH signaling molecules at later stages of neurodevelopment, including axonal and dendritic outgrowth, and synaptogenesis, we performed transcriptomic analyses of rat primary neuron-glia co-cultures derived from PND 1 rat cortex that were cultured for up to 21 days *in vitro* (DIV) [23]. Finally, we also examined the ontogenetic profile of these transcripts in zebrafish larvae since this model has been proposed as an alternative organism for screening the DNT potential of TH active chemical compounds [24,25]. Moreover, zebrafish offer the potential for future studies to integrate molecular effects of TH disruption with physiological and behavioral outcomes.

**Table 1:** Components of the TH signaling system in different species.

Gene name	Gene (isoform) symbol			Function
	human	rat	zebrafish	
L-type amino acid transporter 1	*LAT1 (SLC7A5)*	*Lat1*	*lat1*	TH transporter
L-type amino acid transporter 2	*LAT2*	*Lat2*	*lat2*	TH transporter
Monocarboxylate transporter 8	*MCT8 (SLC16A2)*	*Mct8*	*mct8*	TH transporter
Organic anion transporting protein 1C1	*OATP1C1*	*Oatp1c1*	*oatp1c1*	TH transporter
Deiodinase 2	*DIO2*	*Dio2*	*dio2*	TH activating enzyme
Deiodinase 3	*DIO3*	*Dio3*	*dio3a dio3b*	TH inactivating enzyme
Thyroid hormone receptor alpha	*THRA1 THRA2*	*Thra1 Thra2*	*thraa thrab*	TH receptor
Thyroid hormone receptor beta	*THRB*	*Thrb*	*thrb*	TH receptor
Nuclear Receptor Corepressor 1	*NCOR1*	*Ncor1*	*ncor1*	Corepressor of TH receptor
Krueppel-like factor 9	*KLF9*	*Klf9*	*klf9*	TH target gene
Myelin basic protein	*MBP*	not studied*	*mbpa*	TH target gene
Myelin-Associated Oligodendrocyte Basic Protein	not studied*	*Mobp*	not studied*	TH target gene
Hairless	*HR*	*Hhr*	*hr*	TH target gene

*respective genes were not studied in the indicated species.

Collectively, the findings from this study indicate that while all of these models express a fairly comprehensive transcriptome of TH signaling molecules, their expression does not correlate with functionality of TH signaling studied by target gene expression responses to TH.

## Materials and methods

### Animals

Studies involving primary rat cortical cell cultures and transcriptomic analyses of rat cortical tissues performed at the University of California-Davis (UC Davis) were approved by the UC Davis Institutional Animal Care and Use Committee (Public Health Services (PHS)/National Institutes of Health (NIH) Assurance #A3433-01; protocol 20541). Studies involving rat neurospheres and transcriptomic analyses of whole rat brains performed at the IUF in Düsseldorf were approved by the State Agency for Nature, Environment and Consumer Protection North Rhine-Westphalia (LANUV) in Germany (license number 84–02.05.40.14.140). Timed pregnant Sprague Dawley rats were purchased by UC Davis investigators from Charles River Laboratories (Hollister, CA, USA) whereas timed pregnant Wistar rats were purchased by IUF investigators from Charles River Laboratories (Sulzfeld, Germany). Pregnant females were housed individually in plastic cages with corn cob bedding in a temperature controlled room (22 ± 2 ^o^C) on a 12 hr light-dark cycle. Food and water were provided *ad libitum* to the dams.

Studies involving zebrafish were performed at UC Davis in accordance with protocols approved by the UC Davis Institutional Animal Care and Use Committee (PHS/NIH Assurance #A3433-01; protocols 17645 and 19391). Adult wildtype zebrafish (5D) were originally obtained from the Sinnhuber Aquatic Research Laboratory (SARL) at Oregon State University (Corvallis, OR, U.S.) and subsequent generations were raised at UC Davis. Adult zebrafish were maintained in a 14 h light (~850 lux)/10 h dark photoperiod (Harper and Lawrence, 2016) in water at a temperature of 28.5 ± 0.5 °C, pH of 7.2 ± 0.4, and conductivity of 700 ± 100 μS. Adult fish were fed twice daily with live *Artemia nauplii* (INVE Aquaculture, Inc., Salt Lake City, UT, USA) and a mixture of the following commercial flake foods: Zeigler Zebrafish Granule (Ziegler Bros, Inc. Gardners, PA, USA), Spirulina flake (Zeigler Bros, Inc.), Cyclopeeze (Argent Aquaculture, Redmond, WA, USA), and Golden Pearl (Brine Shrimp Direct, Ogden, UT, USA). Adult zebrafish were spawned naturally in groups of 8–10 fish; embryos were collected and staged following fertilization as previously described (Kimmel et al., 1995).

### Zebrafish embryo sampling

Zebrafish embryos and larvae were raised in embryo medium (15 mM NaCl, 0.5 mM KCl, 1.0 mM MgSO_4_, 150 μM KH_2_PO_4_, 50 μM Na_2_HPO_4_, 1.0 mM CaCl_2_, 0.7 mM NaHCO_3_) in 30 mm tissue culture dishes placed in a 28.5 °C incubator with a 14 h light/10 h dark photoperiod. Zebrafish embryos were collected for transcriptomic analyses at 18, 24, 72, and 120 hours post fertilization (hpf). Groups of 30 embryos were pooled and stored in 1 mL RNAlater (Thermo Fisher Scientific) at 4 °C for 24 h and then at -20 °C until RNA was extracted. Three biological replicates were collected at each time point with embryos spawned from different adult pairs. In order to determine the change in expression induced by TH exposure, zebrafish embryos were treated with embryo medium supplemented with T4 (Sigma) or T3 (Sigma) at 10, 30, or 100 nM in 6-well tissue culture plates (BD Falcon, Corning) beginning at 6 hpf. Twenty embryos were treated in each well and pooled at the time of sample collection. Embryos were collected in RNAlater (ThermoFisher Scientific), stored at 4 °C for 24 h and then at -20 °C until RNA was extracted.

### Cell culture

#### Rat cortical cell cultures (rCCC)

Primary cultures of dissociated rat cortical neuron-glia co-cultures were prepared and maintained as previously described [27]. Briefly, the neocortices of postnatal day (PND) 0–1 rat pups (males and females from the same litter were pooled) were enzymatically dissociated and plated in 24-well tissue culture plates (Sarstedt) coated with poly-L-lysine (molecular weight 300,000, Sigma). Cultures were maintained in NeuralQ Basal Medium (MTI-Global Stem) supplemented with 2% GS21 Neural Supplement (MTI-GlobalStem) and 2 mM Glutamax (Invitrogen) in the absence of serum. At DIV 4, cytosine-D-arabinofuranoside (Sigma) was added to the culture medium at a final concentration of 5 μM. Half of the medium in each well was replaced with fresh media weekly. In experiments where TH concentration was altered, cultures were maintained in serum-free NeuralQ Medium supplemented with 2 mM Glutamax, and a modified NS21 formulation [28] with 0.3, 3 or 30 nM of T3 (Sigma) or T4 (Sigma); these TH concentrations were kept constant throughout the experiment. In each experiment, 3 wells were set up per treatment condition. Each experiment was performed 3 times using cultures derived from independent dissections.

#### Rat and human neural progenitor cell cultures

Normal human neural progenitor cells (hNPC, gestational week 16–19) were purchased from Lonza Verviers SPRL. Experiments with hNPC were approved by the Ethics Committee of the Heinrich-Heine-University, Düsseldorf. Rat neural progenitor cells (rNPC) were prepared as described previously [29] from Wistar Rats (Charles River, Sulzfeld, Germany) on postnatal day 1, which is a developmental stage comparable to that of the hNPC [20]. Human and rat NPC were cultured in proliferation medium consisting of 3:1 DMEM (Life Technologies) and Hams F12 (Life Technologies) media supplemented with 2% B27 (Life Technologies), 20 ng/mL epidermal growth factor (EGF, Life Technologies), 20 ng/mL human (hNPC) or 10 ng/mL rat (rNPC) recombinant human fibroblast growth factor (FGF, R&D Systems), and 1% penicillin and streptomycin (Pan-Biotech). NPC were grown in a humidified incubator at 37 °C with 5% CO_2_ in suspension culture as neurospheres and half of the medium was exchanged thrice a week. Neurospheres were mechanically passaged using a tissue chopper 2–3 days before starting proliferation or differentiation experiments.

For proliferation experiments, 0.3 mm neurospheres were treated with proliferation medium containing either vehicle (0.01% EtOH/HCl, which is a 1:1 mixture of 96% ethanol (EtOH) and 1 M HCl obtained from Carl Roth GmbH), T3 (Sigma) or T4 (Sigma) at 30 fM, 30 pM or 30 nM for 3 d in 24-well plates (Sarstedt). In each experiment, triplicates of 15 neurospheres per well were used per treatment condition. Differentiation was initiated by withdrawing growth factors and plating 0.3 mm neurospheres onto poly-D-lysine (PDL)/laminin (Sigma-Aldrich) coated 24-well plates in 1 mL differentiation medium (3:1 Dulbecco´s modified Eagle medium and Hams F12 supplemented with 1% N2 (Invitrogen GmBH) and 1% Penicillin/Streptomycin (Pan-Biotech, Aidenbach, Germany) containing either vehicle (0.01% EtOH/HCl), T3 or T4. In each experiment triplicates of 15 neurospheres per well were plated per treatment condition. Each proliferation and differentiation experiment was performed at least three times independently.

### RNA extraction and cDNA synthesis

#### Rat cortical cell cultures (rCCC) and rat cortical tissue

RNA was isolated from primary rat cortical cell cultures on DIV 2, 7 and 21 using TRIzol Reagent (ThermoFisher Scientific) according to the manufacturer’s protocol. RNA was isolated from rat cortical tissue (neocortex) collected on PND 0/1 and 7 from euthanized rat pups. Neocortices from male and female pups of the same litter were pooled, and samples were collected from 3 independent litters (n = 3 biological replicates for each time point). Harvested tissues were placed in RNAlater solution (Ambion/ThermoFisher Scientific) overnight at 4 ^o^C and stored at -20 ^o^C until further processed. Cortical tissues were thawed on ice, and then homogenized in TRIzol Reagent. RNA was isolated from homogenates according to the manufacturer’s protocol. Following extraction, RNA concentration was determined using a NanoDrop ND-1000 spectrophotometer (NanoDrop Technologies Inc.). Nucleic acid purity was determined based on the ratio of absorbance at 260 nm to 280 nm (A260/A280 of 1.8–2.2 was considered acceptable), and the absence of genomic DNA contamination was determined by visualization on an agarose gel stained by SYBR Green nucleic acid gel stain (ThermoFisher Scientific). RNA was reverse transcribed at a concentration of 1 μg of RNA per 20 μL reaction, using the Superscript VILO cDNA synthesis kit (Invitrogen) according to the manufacturer’s instructions.

#### Zebrafish

To extract RNA, zebrafish were removed from RNAlater and homogenized in 350 μL of RLT buffer in the Qiagen RNeasy kit (Qiagen) with 1 mm glass beads using the Bullet Blender (Next Advance, Troy, NY). Homogenates were transferred to 1.5 mL Eppendorf tubes and spun in a tabletop centrifuge at maximum speed (21000 x g) for 3 min. The supernatant was transferred to 2 mL Eppendorf tubes and RNA was extracted using the Qiagen RNeasy kit and an automated QIAcube robotic workstation (Qiagen) according to the manufacturer’s instructions. RNA concentration was determined using a NanoDrop ND-1000 spectrophotometer (NanoDrop Technologies Inc.). Nucleic acid purity was determined based on the ratio of absorbance at 260 nm to 280 nm (A260/A280 of 1.8–2.2 was considered acceptable), and the absence of genomic DNA contamination was determined by visualization on an agarose gel stained by SYBR Green nucleic acid gel stain (ThermoFisher Scientific). RNA was reverse transcribed, at a concentration of 1 μg of RNA per 20 μL reaction, using Superscript VILO cDNA synthesis kit (Invitrogen) according to the manufacturer’s instructions.

#### Rat and human neural progenitor cells

NPC were lysed after 3 days of proliferation or after 3 or 5 days of differentiation. RNA was extracted using RNeasy Mini Kit (Qiagen) according to the manufacturer’s instructions. The maximal volume of NPC sphere RNA was transcribed into cDNA using the QuantiTect Reverse Transcription Kit (Qiagen).

#### Human brain

Human brain total RNA sample was purchased from Clontech (pooled male/female, GW 20–33, catalog #636526) and stored at -80 °C. From every sample, 500 ng RNA were transcribed into cDNA using the QuantiTect Reverse Transcription Kit (Qiagen).

### Generation of PCR standards

#### Rat cortical cell cultures, rat cortical tissues, and zebrafish

PCR standards for each gene of interest (GOI) were generated by amplifying cDNA from the rCCC, rat cortical tissues, or zebrafish embryos using Promega GoTaq Flexi (Promega) according to the manufacturer’s instructions. Amplified cDNA (100 μL) from pooled samples was purified using the QIAquick PCR purification kit (Qiagen) and products were eluted in 50 μL Ultrapure Water. The DNA concentrations were estimated using a NanoDrop ND-1000 spectrophotometer (NanoDrop Technologies Inc.) and used to calculate the number of cDNA molecules/μL. Stock solutions of 1.5 x 10^8^ molecules/μL were generated and stored at -20 °C.

#### Rat and human neural progenitor cells

PCR standards for each GOI were generated from six pooled PCR reaction tubes after performing the RT-PCR (= 90 μL pooled samples). The pooled samples were purified with QIAquick PCR purification kit (Qiagen) and products were eluted with 50 μL EB buffer (included in the kit). The DNA concentration was measured using the NanoQuant plate device in the Tecan infinite M200 Pro reader, and the number of cDNA molecules/μL was calculated. Stock solutions of 1.5 x 10^8^ molecules/μL were generated and stored at -20 °C.

### Quantitative real time PCR (qRT-PCR) using copy number standards

#### Rat cortical cell cultures, rat cortical tissues, and zebrafish

Stock solutions of PCR standards for each GOI in each model were used to generate standard curves ranging from 1.5 x 10^2^ molecules/μL to 1.5 x 10^7^ molecules/μL. qRT-PCR was performed on a laser 7900 HT FAST platform (Applied Biosystems, Foster City, CA, USA). Standard curves were run in parallel with cDNA samples with unknown transcript concentrations from rCCC, rat cortical tissues, and zebrafish. Samples were run in a 384-well format with 12 μL of PCR mix per well. PCR mix consisted of 6 μL 2X SYBR Green Power Master Mix (Life Technologies) containing a hot start polymerase that avoids primer dimer and unspecific product formation, 0.25 μL of 10 mM stock forward primer (200 nM final concentration), 0.25 μL of 10 mM stock reverse primer (200 nM final concentration), 1 μL cDNA (~50 ng/reaction), and 4.5 μL UltraPure water. The PCR amplification protocol was as follows: 50 °C for 2 min, 95 °C for 10 min, and 40 cycles of 95 °C for 15 s, 60 °C for 30 s, and 72 °C for 30 s. Each sample was run in triplicate. The cycle threshold (Ct) values obtained for each sample of the standard curves were used to calculate the copy number/μL of each GOI for all unknown target samples. The expression levels (as copy number/μL) were normalized to the expression of a reference gene, and the data are presented as the mean copy number/10,000 reference gene (*ppia or pgk1* for rCCC, *ppia* for rat cortical tissue and β-actin for zebrafish) of three biological replicates. A target gene was considered to be quantifiable if the ratio of the copy number target gene to the copy number for β-actin × 1,000 exceeded 0.001. This method allows comparison of expression levels and developmental patterns of transcript expression across models of different species. All primers used are presented in S1 Table in the supplementary materials.

#### Rat and human neural progenitor cells/Human brain total RNA samples

Stock solutions of PCR standards for each GOI for each model were used to generate standard curves ranging from 1.5 x 10^2^ molecules/μL to 1.5 x 10^7^ molecules/μL. qRT-PCR was performed using the Rotor-Gene Q instrumentation (Qiagen). Standard curves were run in parallel with cDNA samples with unknown transcript concentrations from rat and human neurosphere cultures. The PCR mix consisted of 7.5 μL of PCR Master Mix SYBR FAST (Qiagen) containing a hot start polymerase that avoids primer dimer and unspecific product formation, 2.5 μL solutions of each primer (stock concentration 4 μM) and 2.5 μL of cDNA (1:2.5 diluted). Samples were initially incubated for 7 min at 95 °C to activate the DNA polymerase. The conditions for PCR amplifications were 47 cycles of 10 s at 95 °C for denaturation, 35 s at 60 °C for primer annealing, elongation and fluorescence detection. The cycle threshold (Ct) values obtained for each sample of the standard curves were used to calculate the copy number/μL of each GOI for all unknown target samples. The expression levels (as copy number/μL) were normalized to the expression of the reference gene β-actin. The data are presented as the mean copy number/10,000 β-actin of three biological replicates. All primers used are presented in S1 Table in the supplementary materials.

### Real time PCR (RT-PCR) using ddCT evaluation

Primer efficiencies were calculated from the slope of the standard curves using the following equation:

$$PAE=10^{-\frac{1}{slope}}-1$$ (1)

This calculation determines the percentile amplification efficiency (PAE), which is between 0 and 1 [30]. The CT values for GOI and reference genes were corrected by multiplying them by their calculated PAE. PAEs for primer pairs used in experiments with human NPC were between 0.917 (hHR) and 1.116 (hTHRa1), in experiments with rat NPC between 0.857 (rlat2) and 1.069 (rthra1) and for primer pairs used in experiments with zebrafish were between 0.86 (thrαa) and 1.10 (lat1). From the PEAs, dCT was calculated for each sample by subtracting the corrected CT value of the reference gene from the one of the GOI. The ddCT values were determined by subtracting the dCT mean of the pooled proliferation/earliest time-point/solvent control samples of all three independent experiments from the dCT value of each sample. 2^-ddCT^ was calculated for each sample. 2^-ddCT^ values were pooled for each experimental group and the SEM was calculated. All sample and standard curve CT values as well as standard curve equations are provided in the supporting information file S1 Appendix.

### Statistical analysis

Statistical analyses were performed with GraphPad Prism 6. Transcript levels of TH signaling components and TH-responsive genes were compared as copy numbers/10,000 reference gene between *in vitro* samples and the respective *in vivo* brain samples and as ddCT between PND 0–1 and PND 7 rat cortex using unpaired student’s t-test with Welsh’s correction not assuming equal SDs. P<0.05 was considered as significant. Comparisons of mRNA levels across three different conditions (proliferating or 3 or 5 days differentiating NPC; three different time-points for rCCC, zebrafish; TH treatments with increasing concentrations) were evaluated as ddCT and compared to proliferating neurospheres, the earliest time-point (rCCC, zebrafish) or the vehicle controls using one-way ANOVA with Bonferroni’s multiple comparison test. P<0.05 was considered significant.

## Results

**Fig 3:**
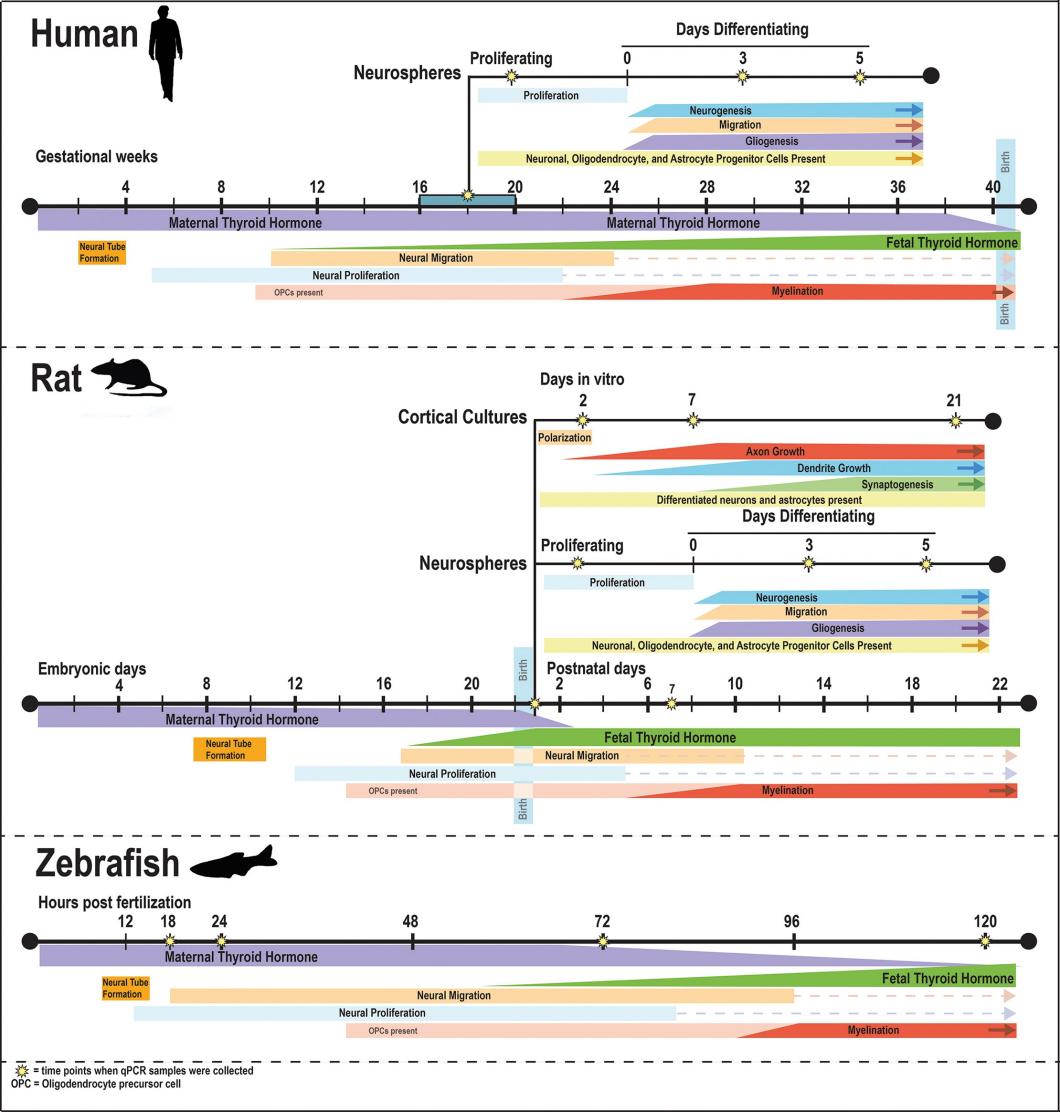
Timing of key neurodevelopmental processes in human, rat, and zebrafish in comparison to the human and rat *in vitro* models of neurodevelopment used to compare developmental expression profile of genes encoding TH signaling molecules. The approximate onset and duration of major neurodevelopmental processes are depicted for each of the *in vivo* and *in vitro* models of neurodevelopment included in the transcriptional profiling of TH signaling genes. In the human, 40 weeks of gestation corresponded to 22 embryonic days and 22 postnatal days in the rat, and 120 hours post fertilization (5 days) in the zebrafish. The developmental time line for the *in vitro* models branch from *in vivo* time scales at the point when tissue was harvested for *in vitro* cultures; however, the subsequent timeline of neurodevelopment differs between *in vitro* models. Yellow stars indicate the time points when gene expression was analyzed. [20,31–37].

**Fig 4:**
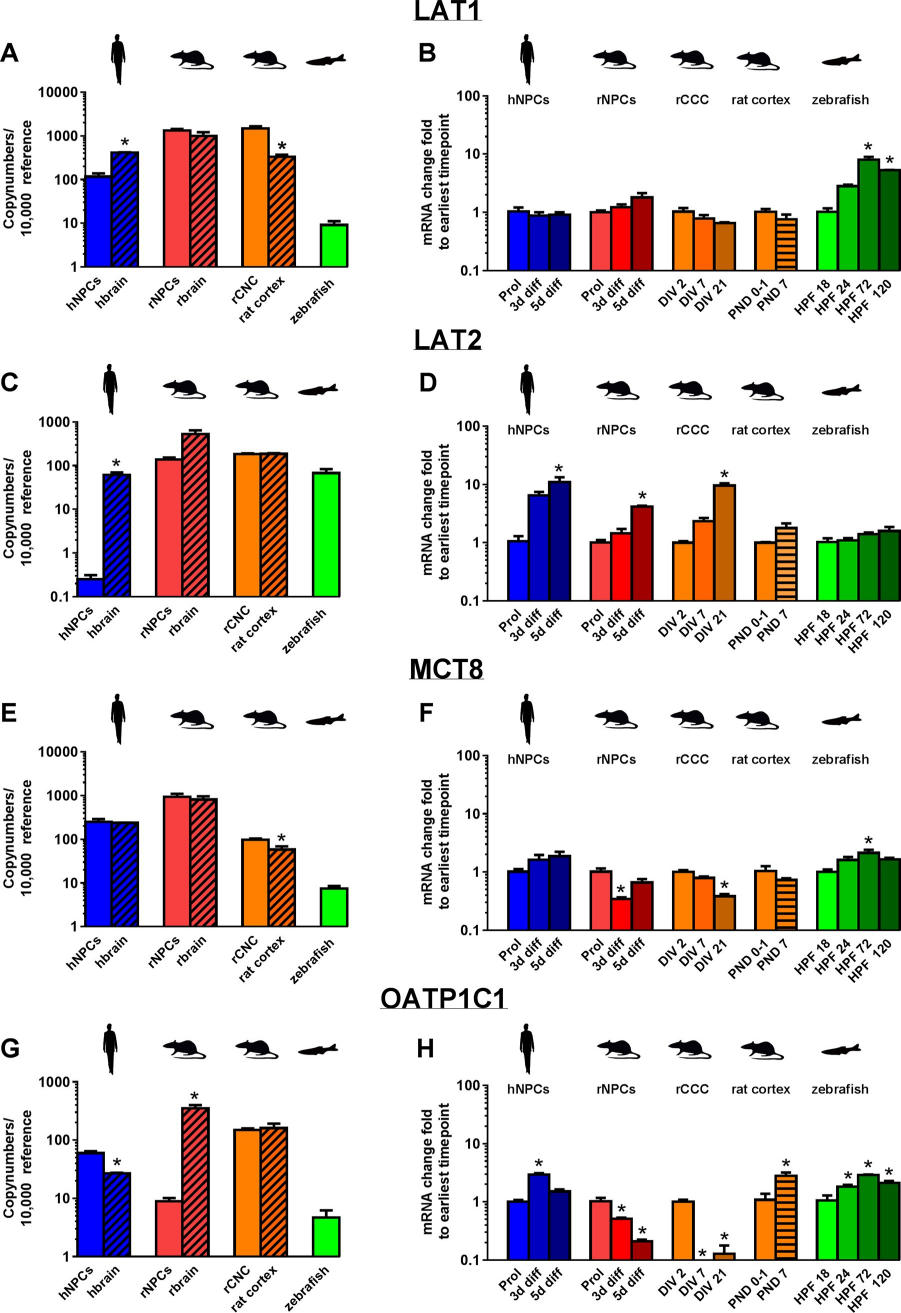
Expression of mRNA encoding thyroid hormone transporters in models of neurodevelopment. Copy numbers of transcripts encoding LAT1 (A), LAT2 (C), MCT8 (E) and OATP1c1 (G) were determined by quantitative real time (qRT-PCR) in proliferating hNPC and rNPC, whole brain samples from human (gestational week 20–33) and rat (postnatal day, PND 1), rCCC at DIV 2, rat cortical tissue (PND 1), and zebrafish (18 hpf). Data are presented as copy numbers per 10,000 copies of reference gene. Reference genes used in these studies were: (1) β-actin for hNPC, rNPC, human brain, rat brain, and zebrafish; and (2) ppia for rCCC and rat cortical tissue. The change in expression over time of genes encoding LAT1 (B), LAT2 (D), MCT8 (F) and OATP1c1 (H) in hNPC, rNPC, rCCC, rat cortical tissue, and zebrafish represented as the fold-change from the earliest time point investigated in each model as determined using the ddCT method. Data are presented as the mean + SE (n = 3 biological replicates). In graphs showing copy number, * indicates significant difference between the in vitro model and its corresponding in vivo sample at p<0.05 as determined by student’s t-test. In graphs showing ddCT, * indicates significant difference relative to earliest time point investigated in the model at p<0.05 as determined by one-way ANOVA with Bonferroni multiple comparison test, with the exception of data for rat cortical tissues, which consisted of only two time points and, thus, was analyzed using student’s t-test.

**Fig 5:**
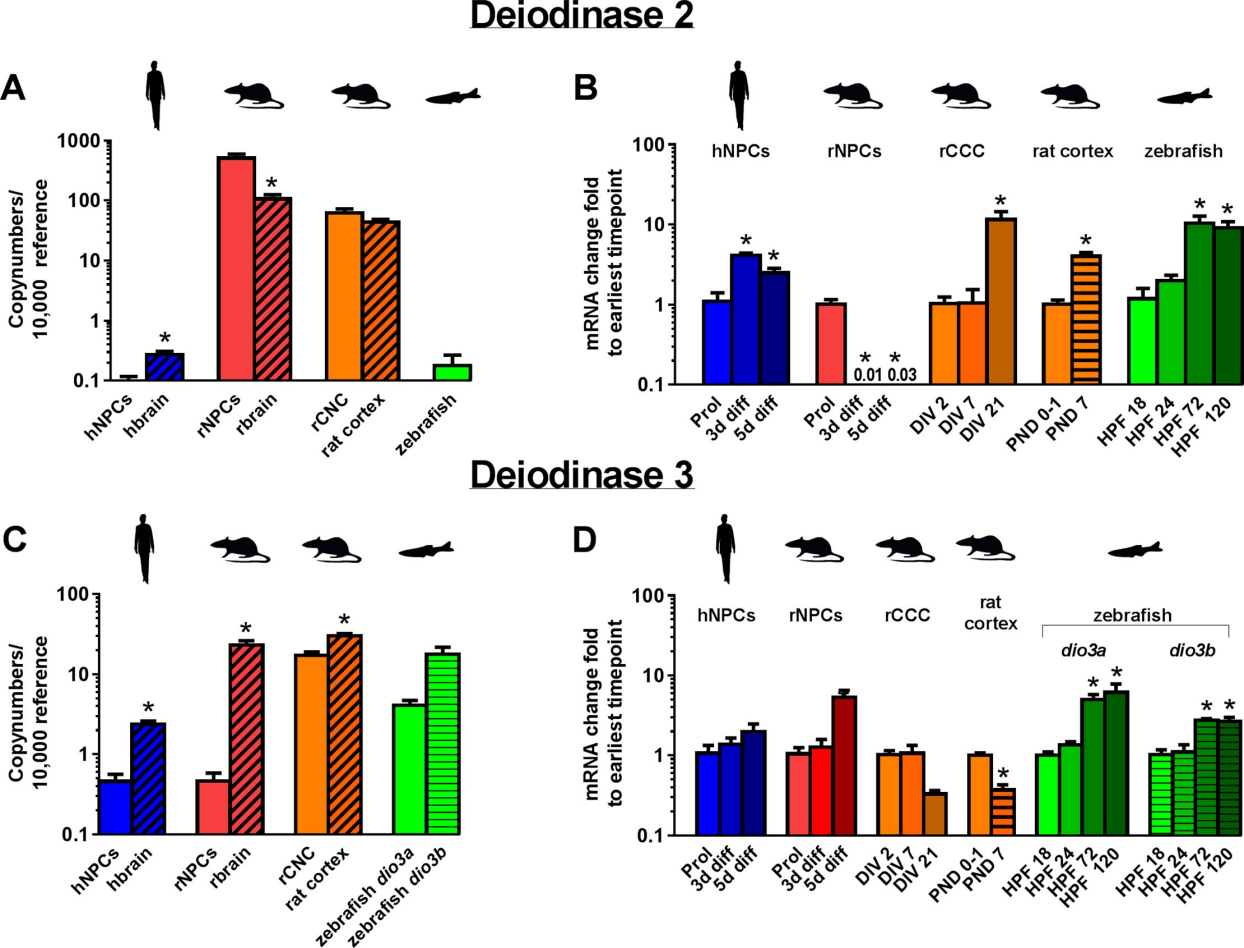
Expression of mRNA encoding deiodinases in models of neurodevelopment. Copy numbers of transcripts for Dio2 (A) and Dio3 (C) were determined by quantitative real time (qRT-PCR) for proliferating hNPC and rNPC, whole brain tissue from human (gestational week 20–33) and rat (PND 1), rCCC at DIV 2, rat cortical tissue (PND 1), and zebrafish (18 hpf). Data are presented as copy numbers per 10,000 copies of reference gene. Reference genes used in these studies were: (1) β-actin for hNPC, rNPC, human brain, rat brain, and zebrafish; and (2) ppia for rCCC and rat cortical tissue. The change in expression over time of Dio2 (B) and Dio3 (D) is shown for hNPC, rNPC, rCCC, rat cortical tissue, and zebrafish as the fold-change from the earliest time point investigated in each model as determined using the ddCT method. Data are presented as the mean + SE (n = 3 biological replicates). In graphs showing copy number, * indicates significant difference between the in vitro model vs. the corresponding in vivo model at p<0.05 as determined by student’s t-test. In graphs showing ddCT, * indicates significant difference relative to the earliest time point investigated in each model at p<0.05 as determined by one-way ANOVA with Bonferroni multiple comparison test, with the exception of data from rat cortical tissue, which consisted of only two time points and, thus, was analyzed using student’s t-test.

**Fig 6:**
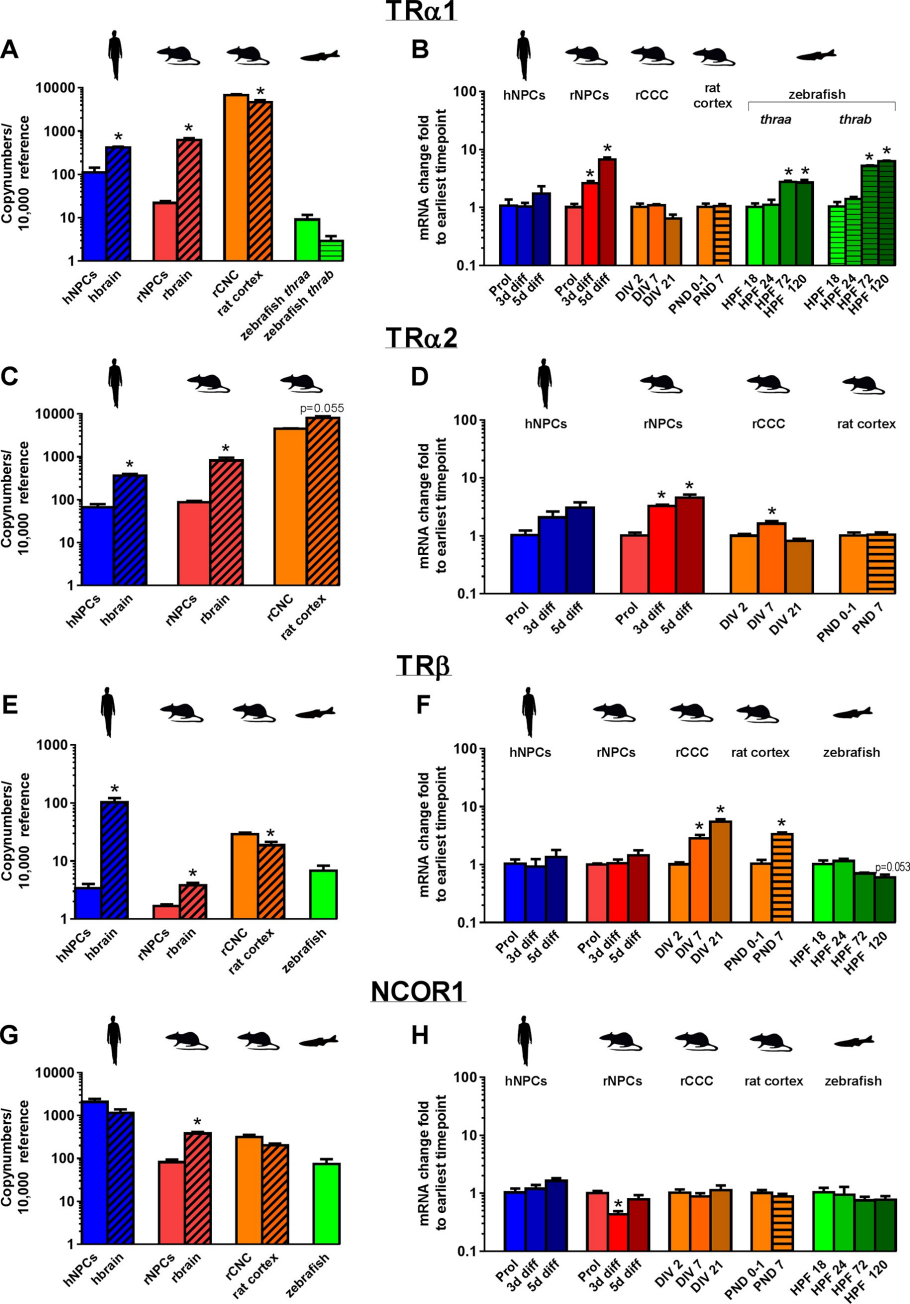
Expression of mRNA encoding specific thyroid hormone receptor (THR) subunits and nuclear corepressor 1 (NCOR1) in models of neurodevelopment. Copy numbers of THRα1/Thrα1/thrαa/thrab (A), THRα2/Thrα2 (C), THRβ/Thrβ/thrβ (E), and NCOR1/Ncor1/ncor1 (G) mRNA were determined by quantitative real time (qRT-PCR) for proliferating hNPC and rNPC, whole brain tissue from human (gestational week 20–33) and rat (PND 1), rCCC at DIV 2, rat cortical tissue (PND 1), and zebrafish (18 hpf). Data are presented as copy numbers per 10,000 copies of reference gene. Reference genes used in these studies were: (1) β-actin for hNPC, rNPC, human brain, rat brain, and zebrafish; and (2) ppia for rCCC and rat cortical tissue. The change in gene expression over time of transcripts encoding THRα1 (B), THRα2 (D), THRβ (F) and NCOR1 (H) is shown for hNPC, rNPC, rCCC, rat cortical tissue, and zebrafish as the fold-change from the earliest time point investigated in each model, as determined using the ddCT method. Data are presented as the mean + SE (n = 3 biological replicates). In graphs showing copy number, * indicates significant difference between in vitro models and their corresponding in vivo models at p<0.05 as determined by student’s t-test. In graphs showing ddCT, * indicates significant difference relative to earliest time point investigated in each model at p<0.05 as determined by one-way ANOVA with Bonferroni multiple comparison test, with the exception of data from rat cortical tissue, which consisted of only two time points and, thus, was analyzed using student’s t-test.

**Fig 7:**
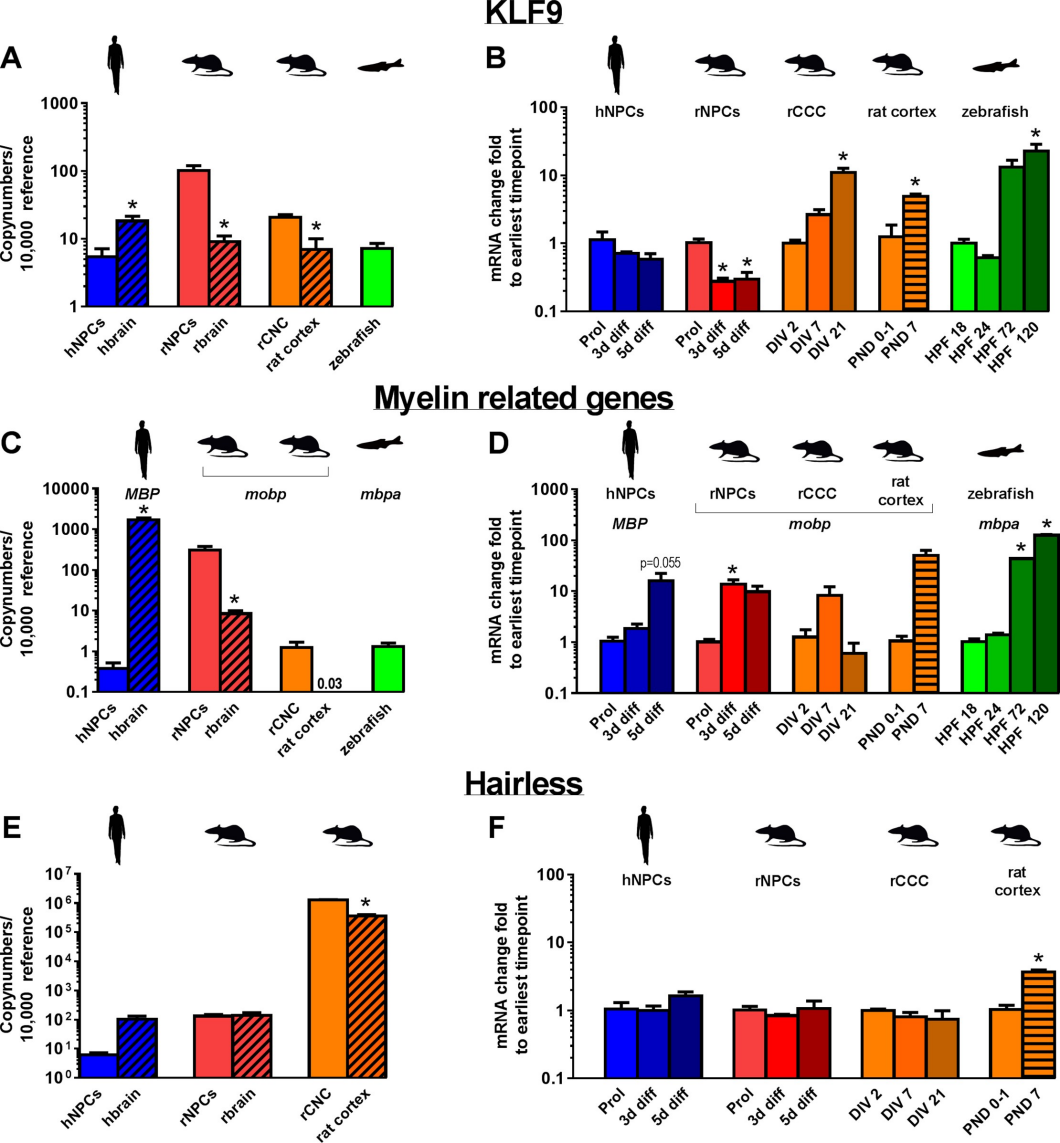
Expression of mRNA encoding TH-responsive genes in models of neurodevelopment. Copy numbers of mRNA encoding KLF9 (A), MBP/MOBP (C), and HR (E) were determined by quantitative real time (qRT-PCR) for proliferating hNPC and rNPC, whole brain tissue from human (gestational week 20–33) and rat (PND 1), rCCC at DIV 2, rat cortical tissue (PND 1), and zebrafish (18 hpf). Data are presented as copy numbers per 10,000 copies of reference gene. Reference genes used in these studies were: (1) β-actin for hNPC, rNPC, human brain, rat brain, and zebrafish; and (2) ppia for rCCC and rat cortical tissue. The change in gene expression over time of KLF9/Klf9/klf9 (B), MBP/Mobp/mbpa (D), and HR/Hr/hr (F) is shown for hNPC, rNPC, rCCC, rat cortical tissue, and zebrafish as the fold-change from the earliest time point investigated in each model, as determined using the ddCT method. Data are presented as the mean + SE (n = 3 biological replicates). In graphs showing copy number, * indicates significant difference between the in vitro model and the corresponding in vivo model at p<0.05 as determined by student’s t-test. In graphs showing ddCT, * indicates significant difference relative to the earliest time point investigated in each model at p<0.05 as determined by one-way ANOVA with Bonferroni multiple comparison test, with the exception of data from the rat cortex, which consisted of only two time points and, thus, was analyzed using student’s t-test.

The ontogenetic profile of transcripts encoding TH transporters, deiodinases, TH receptors, and TH-responsive genes was determined for three *in vitro* models of neurodevelopment–human neurospheres (hNPC), rat neurospheres (rNPC), and rat cortical cell cultures (rCCC)–and in the corresponding *in vivo* models from which these cultures were derived, e.g., human brain (hbrain), rat brain (rbrain), and rat neocortex, respectively. The same transcripts were also evaluated in embryonic/larval zebrafish (Fig 2). Multiple time points were selected for transcriptomic analyses within each model to cover different stages of neurodevelopment (Fig 3). The hNPC and rNPC models mimic the neurodevelopmental processes of proliferation, migration, and fate decision—neurogenesis and gliogenesis. Primary rCCC dissociated from pooled neocortices of male and female rat pups at PND 1 were used to examine transcript expression during neuronal polarization, axon outgrowth, dendritic arborization and synaptogenesis. These processes are present *in vitro*, yet in a simplified manner compared to *in vivo*. Transcripts were quantified in zebrafish at 18, 24, 72, and 120 hours post fertilization (hpf), which encompasses both early neurodevelopmental stages, such as neural proliferation and neural migration (18 hpf) and later stages of neurodevelopment such as synaptogenesis and the development of mature myelinated neuronal networks (120 hpf). In larval zebrafish, the CNS is a predominant region of early expression of TH signaling genes; however, it is important to note that whole zebrafish larvae were analyzed in these experiments thus changes in gene expression incorporate changes within the whole larvae. To compare expression levels and developmental patterns of transcript expression across these models, the absolute gene copy number of each gene was determined in proliferating NPC and at the earliest time point investigated in rCCC and zebrafish embryos. These values were compared to copy numbers of each target gene in the corresponding *in vivo* model. In NPC models, the relative expression of each gene in differentiating cultures was compared to its expression in proliferating NPC, and in rCCC and zebrafish, the relative expression over time was determined using the ddCT method to compare expression at later time points to the earliest time point investigated in the model. The mean ± SE values for the copy number and ddCT data presented as bar graphs in Figs 4–7 are provided in S2 and S3 Tables in the supporting information.

### TH transporters

The absolute copy numbers of mRNA encoding the TH transporters L-type amino acid transporter 1 (*LAT1; SLC7A5*), *LAT2 (SLC7A8)*, monocarboxylate transporter 8 (*MCT8; SLC16A2*), and organic anion transporter 1C1 (*OATP1C1; SLCO1C1*) were determined at the earliest developmental time examined in each model system (Fig 4A, 4C, 4E and 4G) and used as the reference point for determining relative changes in transcript levels as development proceeded (Fig 4B, 4D, 4F and 4H). Transcript for *LAT1* was expressed in all the models investigated, but at differing levels with expression of *Lat1* in the rat model one to two orders of magnitude higher than *LAT1* in human and *lat1* in the zebrafish models. While expression of *LAT1* in hNPC was significantly lower than in human brain (hbrain), and expression of *Lat1* in rCCC at DIV 2 was significantly higher than in rat cortical tissues at PND 1, the absolute copy numbers for *LAT1*/*Lat1* differed by less than one order of magnitude between the *in vitro* models and the *in vivo* tissues from which they were derived (Fig 4A, S2 Table). The relative expression of *LAT1*/*Lat1* over time did not change in the human and rat models; however, in the zebrafish model, *lat1* expression increased as development proceeded (Fig 4B).

Transcripts encoding LAT2 were expressed at similar levels in all *in vitro* models with the exception of proliferating hNPC, which had a very low copy number of *LAT2*. *Lat2* expression in the rat *in vitro* models (rNPC and rCCC) was similar to that observed in the corresponding *in vivo* tissues, while the copy number of *LAT2* in hNPC was more than two orders of magnitude lower than in hbrain (Fig 4C). However, *lat2* expression in zebrafish at 18 hpf was similar to *LAT2* levels in hbrain. Relative expression of *LAT2*/*Lat2* increased with increasing developmental age in hNPC, rNPC and rCCC, while no changes in *Lat2/Lat2* expression were observed in rat cortical tissues or zebrafish (Fig 4D).

The TH transporter *MCT8* was expressed at the mRNA level in all models, with rNPC and rbrain exhibiting the highest copy numbers, followed by hNPC and hbrain and then rCCC and rat cortical tissues (Fig 4E). At 18 hpf, the zebrafish expressed *mct8* at levels that were one to two orders of magnitude lower than in human and rat models. Comparing *in vitro* models to corresponding *in vivo* models, *MCT8/Mct8* expression was similar; however, there was a small but significantly higher expression of *Mct8* in rCCC relative to rat cortical tissues (Fig 4E). The expression of *MCT8* did not change in hNPC over time; in contrast, expression of *mct8* in the zebrafish increased significantly, while *Mct8* in rNPC and rCCC decreased significantly during development (Fig 4F).

Transcripts encoding OATP1C1 were detected in all models, with the highest level of expression observed in the rbrain, and the lowest, in zebrafish (Fig 4G). Among the three *in vitro* models, rCCC had the highest expression, which was more than an order of magnitude higher than rNPC; whereas hNPC expression of *OATP1C1* was between that observed in the two *in vitro* rat models. Comparison of transcript levels for *OATP1C1/ Oatp1c1* in *in vitro* models versus the corresponding *in vivo* tissues indicated no significant differences between rCCC and rat cortical tissues, significantly higher expression in hNPC *vs*. hbrain, and significantly lower expression in rNPC *vs*. rbrain (Fig 4G). Ontogenetic profiling revealed that mRNA levels of this transporter increased with time in hNPC, rat cortical tissues and zebrafish embryos, but were downregulated with time in rNPC and rCCC (Fig 4H).

### TH deiodinases

Next, we studied expression of the TH deiodinases at the mRNA level (Fig 5). Transcript encoding iodothyronine deiodinase type 2 (DIO2) was detected in all models, but in proliferating hNPC, hbrain, and zebrafish at 18 hpf, expression was below 1 copy/ 10000 reference gene, which was two to three orders of magnitude lower than levels observed in all rat models (Fig 5A). Expression of *Dio2* was similar between rCCC and rat cortical tissues; however, *Dio2* expression was significantly higher in rNPC relative to rbrain. *Dio2* expression was downregulated in rNPC as development proceeded, but transcription of *DIO2/Dio2/dio2* increased with time in hNPC, rCCC, rat cortical tissues, and zebrafish (Fig 5B). Transcripts encoding iodothyronine deiodinase type 3 (DIO3) were also present in all models (Fig 5C). The highest expression was observed in the rat *in vivo* models, rCCC, and zebrafish, which were each one or more orders of magnitude higher than expression in hNPC and rNPC. Zebrafish express two transcript variants of the *dio3* gene, *dio3a* and *dio3b*, both of which were expressed at similar levels. Transcript levels for *DIO3/Dio3* were lower in *in vitro* models relative to their corresponding *in vivo* model, but the most profound difference in copy number was observed between rNPC and rbrain (Fig 5C). Expression of mRNA for *DIO3/Dio3/dio3* over time did not change significantly in hNPC or rCCC, decreased significantly in rat cortical tissues, and increased significantly in rNPC and zebrafish (Fig 5D).

### TH receptors and NCOR1

Fig 6 depicts mRNA copy numbers (Fig 6A, 6C, 6E and 6G) and changes in transcript expression over time (Fig 6B, 6D, 6F and 6H) of the thyroid hormone receptor (THR) isoform *THRα1* (represented by *thrαa* and *thrαb* in zebrafish), *THRα2* (for which no corresponding transcript has been described in zebrafish), and *THRβ*, as well as the nuclear co-repressor (*NCOR1*). The gene encoding the TR receptor isoform α1, *THRa1/Thra1/thraa/thrab*, was expressed in all *in vitro* models, although at differing levels, with the highest copy numbers observed in rCCC, followed by hNPC, and then rNPC. Zebrafish expressed the lowest copy numbers, with copy numbers of both *thrαa* and *thrαb* almost three orders of magnitude lower than was observed for *Thrα1* in rCCC. Comparing *THRα1*/*Thra1* expression in the *in vitro* models to that of corresponding *in vivo* tissues, hNPC and rNPC were both significantly lower than hbrain and rbrain, respectively, while the rCCC had aslightly higher expression than rat cortical tissues (Fig 6A). Analysis of the ontogenetic profile revealed increased expression of *Thrα1* in differentiating rNPC and *thrαa*/*thrαb* in zebrafish with development (Fig 6B). In contrast, *THRα1* in hNPC and *Thrα1* in rCCC and rat cortical tissue did not change significantly over time (Fig 6B).

The mRNA copy number of *Thra*2 in rCCC was almost two orders of magnitude higher than *THRα2* in hNPC or *Thrα2* in rNPC (Fig 6C). Expression in hNPC and rNPC was significantly lower than that observed in their corresponding *in vivo* sources (Fig 6C). Analysis of the ontogenetic expression of *Thra2* revealed increasing expression in differentiating rNPC and rCCC; in contrast, there was no change in relative expression of *THRα2* in hNPC or *Thra2* in rat cortical tissue during development (Fig 6D).

The gene encoding the TR receptor *β*, *THRβ*, was detected in all the models investigated with copy numbers in rCCC one order of magnitude higher than in hNPC and rNPC, which were of the same order of magnitude (Fig 6E). Copy numbers of *thrβ* in zebrafish at 18 hpf were between *THRβ/Thrβ* of rCCC and the NPC models (Fig 6E). *THRβ* expression in hNPC was an order of magnitude lower than in hbrain; in contrast, less pronounced differences were observed between the *in vitro* rat models (rNPC and rCCC) and their *in vivo* counterparts (Fig 6E). Expression of *Thrβ* in rCCC increased over time, mirroring a similar increase in rat cortical tissues (Fig 6F). In contrast, expression of *THRβ* in hNPC, *Thrβ* in rNPC, and *thrβ* in zebrafish did not change over the course of development (Fig 6F).

Transcript encoding NCOR1 was detected in all models investigated (Fig 6G). The highest copy number was observed in hNPC, with lower but similar levels observed in rNPC, rCCC and zebrafish (Fig 6G). While levels of *Ncor1* were significantly lower in rNPC relative to rbrain, across all models, expression levels in the *in vitro* models were of the same order of magnitude as expression levels in their *in vivo* counterparts (Fig 6G). The only difference in expression with development that was observed was a down-regulation of *Ncor1* in differentiating rNPC (Fig 6H).

### TH-regulated neurodevelopmental genes

The genes *kruppel-like factor 9* (*KLF9*), *myelin basic protein* (*MBP*), *myelin-associated oligodendrocyte basic protein* (*MOBP*), and *hairless* (*HR*) are TH-regulated genes important in neurodevelopment [38–41]. Fig 7 depicts the mRNA expression of these genes as absolute copy numbers (Fig 7A, 7C, 7E and 7G) at the earliest time point investigated in each model, and as the fold-change at later developmental times (Fig 7B, 7D, 7F and 7H).

Transcripts encoding KLF9 were detected in all the models investigated. The highest copy number was observed in rNPC, with one order of magnitude lower copy numbers observed in hNPC, rCCC and zebrafish (Fig 7A). Comparing the *in vitro* models to their *in vivo* counterparts, copy numbers of *KLF9* were significantly lower in hNPC than in hbrain, while copy numbers of *Klf9* in rNPC and rCCC were significantly higher than in rbrain and rat cortical tissues, respectively (Fig 7A). Expression of *KLF9* over time was unchanged in hNPC, *Klf9* was downregulated in rNPC but upregulated in rCCC and rat cortical tissues, and *klf9* was upregulated in zebrafish (Fig 7B).

To assess myelin-associated genes, *MBP* was quantified in human models, *Mobp* in rat models, and the zebrafish homolog *mbpa* in zebrafish. *MBP/Mobp/mbpa* was detected in all models, with the highest copy number observed in hbrain, which was approximately three orders of magnitude higher than the copy number measured in hNPC (Fig 7C). In contrast, copy number of *Mobp* in rbrain was significantly lower than rNPC. Expression of *Mobp* in rCCC and rat cortical tissue were both relatively low and were not significantly different from each other (Fig 7C). While *mbp*a was detected in zebrafish at 18 hpf, the copy number was relatively low. The expression of these myelin-associated genes increased with development in all the models; however, only the increased expression of *Mobp* in rNPC and of *mbpa* in zebrafish were statistically significant (Fig 7D).

The gene encoding hairless (HR) was evaluated in human and rat models, but not in zebrafish due to inability to identify an *hr* homolog in zebrafish. The mRNA copy numbers were highest in rCCC and rat cortical tissue, with rCCC only slightly higher than rat cortical tissue (Fig 7E). Expression of *HR* in hNPC and *Hr* in rNPC was several orders of magnitude lower than in rCCC. Expression of *HR* in hNPC was lower than in hbrain, but this difference was not statistically significant; no difference was observed in expression of *Hr* between rNPC and rbrain (Fig 7E). Evaluation of the ontogenetic expression revealed only one model with a significant change over time: *Hr* expression was upregulated with development in rat cortical tissues (Fig 7F).

### Induction of *DIO3*/*Dio3*/*dio3* by exogenous TH

**Fig 8:**
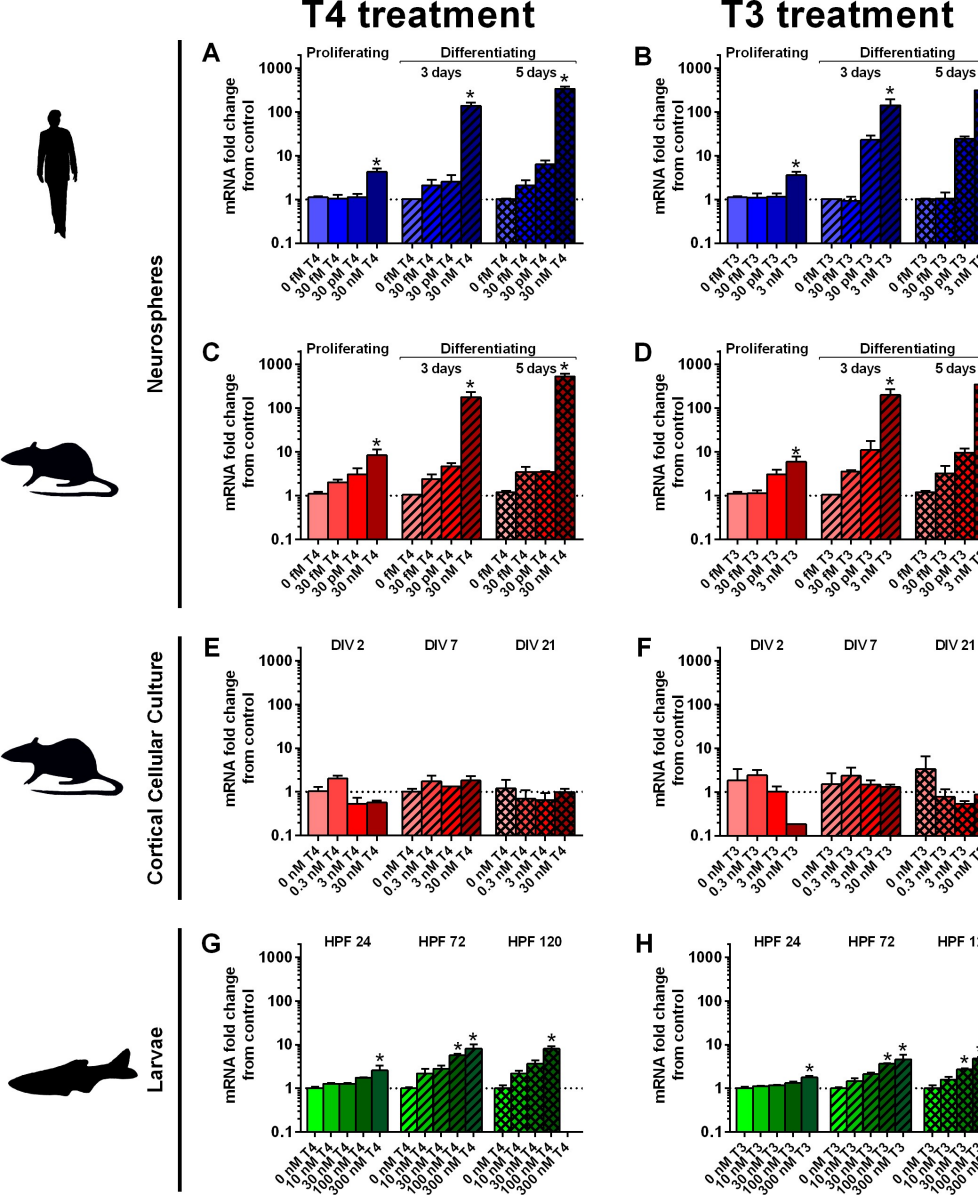
Induction of mRNA encoding Dio3 by exogenous T4 and T3. Transcript levels of DIO3/Dio3/dio3 following treatment with exogenous T4 or T3 were determined by quantitative real time PCR (qRT-PCR) in hNPC (A-B), rNPC (C-D), rCCC (E-F), and zebrafish (G-H) at different developmental time points. Reference genes used in these studies were β-actin (A-D, G-H) and ppia (E-F). Data are presented as the fold-change from vehicle control at the same developmental time point, as determined using the ddCT method. (A-D, G-F) Data are shown as the mean + SE (n = 3 biological replicates); * indicates a significant difference at p<0.05 from the time-matched vehicle control as determined by ANOVA with Bonferroni multiple comparison test. (E-F) Data are shown as the mean + range (n = 2 biological replicates); no statistical evaluation was performed.

To determine whether there were species- or developmental stage-dependent differences in regulation of TH-dependent genes, induction of mRNA encoding DIO3 was examined in hNPC, rNPC, rCCC and zebrafish exposed to varying concentrations of T4 and T3 (Fig 8). T4 (30 nM) and T3 (3 nM) significantly upregulated *DIO3* and *Dio3* in proliferating and differentiating hNPC (Fig 7A and 7B) and rNPC (Fig 8C and 8D), respectively, by two to three orders of magnitude. Neither T4 nor T3 induced *Dio3* expression in rCCC (Fig 8E and 8F). T4 (100 nM) and T3 (100 nM) upregulated *dio3b* in zebrafish (Fig 8G and 8H), although the induction (a maximum of one order of magnitude increase) was less robust than that observed in the NPC models.

## Discussion

Observations from experimental models suggest that TH regulates multiple neurodevelopmental processes, including neurogenesis, dendritic and/or axonal growth, and myelination [16]. Consistent with these preclinical observations, patients with TH deficiency in the CNS due to mutations in the TH transporter MCT8 (Alan-Herndon-Dudley-Syndrome, AHDS) exhibit delayed cortical and cerebellar development and myelination, loss of parvalbumin expression, abnormal calbindin-D28k content and impaired axonal maturation [42–45]. However, critical windows of TH action on specific developmental processes, and mechanisms by which TH disruption interferes with neurodevelopment are not well characterized, and suitable cell models for studying these interactions are largely lacking. As an example, retinoic acid-induced development of NT-2 cells, a common *in vitro* model for studying neuronal development, is not affected by TH despite the fact that this neuronal cell line expresses functional TH transporters [46].

While primary cell cultures are thought to more closely model the *in vivo* situation than cell lines, it would be naïve to expect a one-to-one correlation in either the profile or absolute level of transcripts encoding TH signaling molecules between the *in vitro* models and *in vivo* tissue sources because the *in vitro* models used in this study do not recapitulate the cellular complexity of the *in vivo* situation. For example, NPC used in this study consist of one nestin and sox-2 double positive cell type [47], which is only a fraction of cell types in the developing fetal brain [48]. Similarly, rCCC are limited in their cell type composition compared to rodent postnatal brains *in vivo*. One common difference between our *in vitro* models and the corresponding *in vivo* tissue is the lack of vascular cell types and microglia in the former. The simpler cell composition *in vitro* vs. *in vivo* is important because of the known cell type and brain region specificity of TH signaling [49]. Nonetheless, we sought to determine how closely our *in vitro* systems resembled their *in vivo* counterparts, by comparing mRNA of target genes in our *in vitro* models to the tissues from which our cultures were derived. In addition, we compared gene transcription across species, presenting these data as gene copy numbers generated using product-specific copy number standards to enable valid cross-species evaluations, as previously described [50]. Because the samples were prepared in two different labs with slightly dissimilar protocols, a factor of uncertainty in comparability of the respective gene copy numbers is possibly introduced into the study. However, each of the labs prepared RNA from rat whole brains or brain cortex, respectively. Gene copy numbers for most genes studied were in similar orders of magnitude for both labs, except for TR, which are higher expressed in cortex than in most other brain regions *in vivo* (Dowling et al. J Neurosci 2000). This comparison hints to a fairly reasonable resemblance of qPCR results between the two labs.

Across the different species–human, rat, and zebrafish–and the different brain regions–rat whole brain or cerebral cortex–the genes encoding TH transporters *LAT1*, *LAT2*, *MCT8* and *OATP1C1* are generally expressed, indicating that at least some cell types present in the *in vitro* cultures have the ability to transport TH across their membranes. Compared to the mammalian systems, zebrafish expression of TH transporters is very low. This low TH transporter expression, however, does not seem to limit the zebrafish larvae’s response to TH. In contrast to rat CCC, which have a much higher TH transporter expression than zebrafish larvae, TH-induced *dio3* expression [51,52] is much more robust in zebrafish than in rat CCC. Also, TH transporter expression and TH-dependent *DIO3/Dio3* induction do not correlate between rat and human NPC, indicating that transporter expression is not the limiting factor for TH gene induction in these models. In general, there are differences in TH transporter expression between *in vitro* and *in vivo* models. These differences may reflect the more limited profile of cell types *in vitro*, which lack, for example, microvasculature that expresses *MCT8/10*, *OATP1C1* and *LAT1/2* [17,53,54] during intrauterine development [16,46,53,55,56]. Also of interest, *Mct8* expression is much lower (~ 40-fold less) in the rat cortex than the rat whole brain, which is consistent with the approximate 10-fold higher expression of *Mct8* in rNPC compared to rat CCC. TH transporter expression changes during *in vitro* culture of mammalian cells, reflecting changes in cell differentiation and maturation. Interestingly, zebrafish larvae display a TH transporter induction profile over time that differs from that of the mammalian systems. The reasons and consequences for this species difference are currently enigmatic.

After uptake of circulating T4 in blood by TH transporters expressed on developing brain cells, T4 is converted by DIO2 to the higher TR affinity ligand T3 [26]. Conversely, the TH-inactivating DIO3 metabolizes T4 to reverse T3 and T3 to T2 [57]. Both enzymes are expressed at higher levels in *in vivo* rat compared to human brain, and this difference is maintained in the *in vitro* systems of the respective species. Human NPC display lower *DIO3* expression than human brains, which can be explained by previously published cell type-specific brain expression in neurons [58]. Treatment of NPC cultures with TH, however, strongly induces *DIO3/Dio3* expression in differentiated human and rat NPC by 100- and 500-fold, respectively, while *Dio3* expression is not induced by T3 or T4 in rCCC. Why rCCC do not up-regulate *Dio3* in response to TH is not known as similar cortical cell cultures derived from mouse respond with a 4-fold increase in *Dio3* gene expression upon treatment of 10-day-old cultures with 1 nM T3 for 24 h [52]. In contrast, *DIO2*/*Dio2* was expressed at much higher levels in rat compared to human NPC. Although rat *Dio2* was reported to be mainly expressed in astroglial cells in cortex, hippocampus and cerebellum and in hypothalamic tanocytes of PND 15 neonatal rat brain [59], specific expression in rat NPC had so far not been studied. Assuming a conservation of signaling pathways when cells are transferred from *in vivo* to *in vitro*, there might be a species difference in *DIO2/Dio2* expression in human vs. rat NPC. Interestingly, *dio2* expression in zebrafish larvae is similar to the expression of this gene in human whole brains. Very low expression of *dio2* in zebrafish larvae was reported earlier [60].

TRs are present and functional in the developing brain [49]. The main isoform expressed in mouse neurons and oligodendrocytes is *Trα1*, while *Trβ* is expressed more highly in astrocytes [49,61]. Gene expression data from human and rat neurospheres and corresponding brain samples confirm the higher abundance of *TRα1/Trα1* compared to *TRβ/TRβ* isoforms, and this expression increases during the differentiation of rat NPC. In contrast to human and rat, the zebrafish TR homologues, *traa* and *trab*, are expressed at extremely low levels, and *trb* is more abundant than *tra* isoforms. Despite the low *tr* expression levels in zebrafish larvae, *dio3b* is robustly induced by TH. These data clearly indicate that TR-dependent gene induction by TH is not determined solely by relative expression levels of *tr*. Additional proteins that are part of the TR transcription machinery can regulate TR function [33], for example, the transcriptional co-repressor hairless [62]. Interestingly, *Hr* is expressed three to six orders of magnitude higher in rCCC compared to human or rat NPC, which may explain the lack of TH induction of *Dio3* in these cells compared to the NPC models and the zebrafish.

Previously published TR target genes in brain include *klf9* [63], myelin-related genes [64,65] and *hr* [66]. We observed that these genes are differentially expressed and regulated during cell differentiation and maturation in the absence of TH. Comparing the expression of these genes *in vitro* to the corresponding *in vivo* counterpart is interesting since endogenous TH is present in the *in vivo* situation. Therefore, it is not surprising that *in vivo* samples of hbrain express higher copy numbers of *HR* and *KLF9*, which are considered a readout for general cellular TR activation, than hNPC and four orders of magnitude higher copy numbers of *MBP*, which is a marker of oligodendrocyte maturation. That similar patterns were not observed in the rat models suggests that TH regulation may differ between the developing human and rat brain. These species differences are consistent with previously reported differences in oligodendrocyte differentiation and maturation between mice and humans [65].

In summary, the models examined in this study express multiple TH signaling components but at differing levels. These models also respond differently to TH. The response depends not only on the cell type, but also on the species and stage of cellular maturation. Therefore, when studying chemicals’ interference with TH signaling in developing brain cells, it is important to choose an appropriate model based on the research question being asked. Here, especially *Dio3* expression, but also other TH-responsive genes, such as *Mbp* or *Hr*, can be considered. We recently used the hNPC system to develop an assay for detecting compounds that disrupt TH signaling in oligodendrocytes [65]. Additional work is needed to understand the implications of gene expression of the TH signaling machinery and their phenotypic consequences of chemical disruption of these transcriptomic profiles for the developing brain.

## Supporting information

S1 Table Copy numbers normalized to reference genes for copy number data represented in bar graphs in Figs 4–7 in the main manuscript. (DOCX)

S2 Table Fold change in mRNA expression compared to the earliest time-point (grey) for data represented in bar graphs in Figs 4–7 in the main manuscript. (DOCX)

S3 Table Primer Sequences for human, rat and zebrafish genes used in the manuscript. (DOCX)

S1 Appendix Sample and standard curve CT values as well as standard curve equations are provided for all PCR products. (XLSX)
