# Supplementary material for: Ontogenetic expression of thyroid hormone signaling genes: An in vitro and in vivo species comparison
Source: PLoS One. 2019 Sep 12;14(9):e0221230. doi: 10.1371/journal.pone.0221230 (PMC6742404; doi:10.1371/journal.pone.0221230)
Supplement: S1 Table — (DOCX) [file pone.0221230.s001.docx]

**Supplementary Table 1:** Copy numbers normalized to housekeeping genes for copy number data represented in bar graphs in Figures 4-7 in the main manuscript.

| **Gene symbol** | **Copy numbers/10’000 copies housekeeping gene (mean ± SEM; n=3)** | | | | | | |
| --- | --- | --- | --- | --- | --- | --- | --- |
|  |  | | | | | | |
|  | **Human** | | **Rat** | | | | **Zebrafish**  **(18 hpf)** |
|  | **hNPC (prol)** | **hbrain** | **rNPCs (prol)** | **rbrain (PND1)** | **rCNC (DIV2)** | **Rat cortex (PND0-1)** |  |
| ***Lat1*** | 117 ± 22 | 411 ± 12 | 1338 ± 103 | 998 ± 221 | 1475 ±190 | 335 ± 36 | 9.1 ± 1.2 |
| ***Lat2*** | 0.25 ± 0.06 | 62 ± 9 | 137 ± 16 | 525 ± 118 | 184 ± 8 | 185 ± 7 | 68 ± 9 |
| ***Mct8*** | 252 ± 40 | 237 ± 4 | 947 ± 156 | 816 ±151 | 98 ± 7 | 58 ± 11 | 7.5 ± 0.6 |
| ***Oatp1c1*** | 60 ± 5 | 27 ± 0.6 | 8.9 ± 1.3 | 353 ± 47 | 149 ± 10 | 161 ± 32 | 4.7 ± 0.9 |
| ***Dio2*** | 0.09 ± 0.03 | 0.27 ± 0.04 | 510 ± 86 | 107 ± 19 | 62 ± 10 | 44 ± 5 | 0.18 ± 0.05 |
| ***Dio3*** | 0.46 ±0.10 | 2.4 ± 0.2 | 0.47 ± 0.12 | 23 ± 3 | 17 ± 2 | 30 ± 2 | 4.1 ± 0.4 (*dio3a*)  18 ± 2 (*dio3b)* |
| ***Thra1*** | 112 ± 32 | 422 ± 14 | 22 ± 2 | 622 ± 69 | 6747 ± 355 | 4646 ± 524 | 9.1 ±1.5 (*thraa*)  2.9 ±0.5 (*thrab*) |
| ***Thra2*** | 67 ± 12 | 363 ± 35 | 87 ± 7 | 819 ± 137 | 4519 ±113 | 8013 ± 844 | ---- |
| ***Thrb*** | 3.4 ± 0.6 | 102 ± 19 | 1.7 ± 0.1 | 3.8 ± 0.4 | 29 ± 2 | 19 ± 3 | 6.8 ± 0.9 |
| ***Ncor1*** | 2080 ± 362 | 1141± 258 | 82 ± 12 | 382 ± 32 | 319 ± 37 | 204 ± 19 | 75 ±13 |
| ***Klf9*** | 5.4 ± 1.7 | 18 ± 3 | 102 ± 18 | 9.1 ± 1.9 | 21± 2 | 7.0 ± 3.0 | 7.2 ± 0.8 |
| ***Myelin*** | 0.4 ± 0.1  (*MBP*) | 1696 ± 189 (*MBP*) | 310 ± 66  (*mobp*) | 8.4 ± 1.5  (*mobp*) | 1.2 ± 0.4  (*mobp*) | 0.03 ± 0.01 (*mobp*) | 1.3 ± 0.2  (*mbpa*) |
| ***Hr*** | 6.1 ± 1.2 | 104 ± 28 | 132 ± 16 | 139 ± 34 | 1276509 ± 22464 | 358172 ± 44684 | ---- |

|  | <1 (not present) |
| --- | --- |
|  | 1-10 |
|  | 10-100 |
|  | 100-1000 |
|  | 1000-100000 |
|  | 100000-1000000 |
|  | >10000000 |
