# Supplementary material for: Ontogenetic expression of thyroid hormone signaling genes: An in vitro and in vivo species comparison
Source: PLoS One. 2019 Sep 12;14(9):e0221230. doi: 10.1371/journal.pone.0221230 (PMC6742404; doi:10.1371/journal.pone.0221230)
Supplement: S2 Table — (DOCX) [file pone.0221230.s002.docx]

**Supplementary Table 2:** Change-fold in mRNA compared to the earliest time-point (grey) for data represented in bar graphs in Figures 4-7 in the main manuscript.

|  | | **mRNA change fold to earliest time-point (grey) (mean ± SEM; n=3)** | | | | | | | | | | | | | | | | |
| --- | --- | --- | --- | --- | --- | --- | --- | --- | --- | --- | --- | --- | --- | --- | --- | --- | --- | --- |
|  | | **Human** | | | | | **Rat** | | | | | | | | **Zebrafish** | | | |
| **Gene symbol** | | **hNPC** | | | | | **rNPC** | | | **rCNC** | | | **Rat cortex** | |  |  |  |  |
|  | | **prol** | **3d diff** | **5d diff** | | | **prol** | **3d diff** | **5d diff** | **DIV2** | **DIV7** | **DIV21** | **PND0-1** | **PND7** | **HPF18** | **HPF24** | **HPF72** | **HPF120** |
| ***Lat1*** | | 1.03 ±0.18 | 0.88  ±0.12 | 0.91  ±0.10 | | | 1.00  ±0.07 | 1.22  ±0.15 | 1.80  ±0.34 | 1.02  ±0.16 | 0.79  ±0.11 | 0.65  ±0.02 | 1.01  ±0.12 | 0.76  ±0.16 | 1.02  ±0.15 | 2.81  ±0.19 | 7.99  ±0.94 | 5.34  ±0.01 |
| ***Lat2*** | | 1.06  ±0.24 | 6.53  ±0.90 | 10.97  ±2.32 | | | 1.01  ±0.10 | 1.46  ±0.27 | 4.16  ±0.16 | 1.00  ±0.06 | 2.33  ±0.33 | 9.60  ±0.91 | 1.00  ±0.03 | 1.79  ±0.35 | 1.03  ±0.17 | 1.10  ±0.10 | 1.40  ±0.11 | 1.59  ±0.28 |
| ***Mct8*** | | 1.01  ±0.11 | 1.63  ±0.34 | 1.87  ±0.36 | | | 1.02  ±0.13 | 0.35  ±0.02 | 0.66  ±0.09 | 1.00  ±0.08 | 0.79  ±0.05 | 0.38  ±0.04 | 1.04  ±0.22 | 0.73  ±0.05 | 1.01  ±0.10 | 1.61  ±0.21 | 2.13  ±0.28 | 1.65  ±0.10 |
| ***Oatp1c1*** | | 1.01  ±0.07 | 2.92  ±0.21 | 1.51  ±0.12 | | | 1.02  ±0.15 | 0.51  ±0.02 | 0.21  ±0.01 | 1.01  ±0.08 | 0.07  ±0.01 | 0.13  ±0.05 | 1.09  ±0.29 | 2.80  ±0.39 | 1.06  ±0.24 | 1.83  ±0.14 | 2.91  ±0.06 | 2.11  ±0.18 |
| ***Dio2*** | | 1.11  ±0.30 | 4.10  ±0.30 | 2.49  ±0.36 | | | 1.02  ±0.14 | 0.01  ±0.007 | 0.03  ±0.01 | 1.04  ±0.21 | 1.05  ±0.50 | 11.57  ±2.95 | 1.02  ±0.13 | 4.02  ±0.47 | 1.18  ±0.42 | 2.00  ±0.33 | 10.4  ±2.40 | 9.14  ±1.72 |
| ***Dio3*** | | 1.07  ±0.27 | 1.38  ±0.27 | 1.99  ±0.49 | | | 1.05  ±0.21 | 1.27  ±0.32 | 5.34  ±1.18 | 1.02  ±0.14 | 1.07  ±0.27 | 0.33  ±0.03 | 1.01  ±0.07 | 0.37  ±0.06 | 1.01  ±0.10  (*dio3a*)  1.02  ±0.16  (*dio3b*) | 1.36  ±0.12  (*dio3a*)  1.10  ±0.26  (*dio3b*) | 4.99  ±0.79  (*dio3a*)  2.75  ±0.14  (*dio3b*) | 6.14  ±1.69  (*dio3a*)  2.68  ±0.32  (*dio3b*) |
| ***Thra1*** | | 1.07  ±0.30 | 1.04  ±0.16 | 1.74  ±0.58 | | | 1.02  ±0.13 | 2.61  ±0.25 | 6.75  ±0.55 | 1.02  ±0.14 | 1.09  ±0.04 | 0.64  ±0.11 | 1.02  ±0.15 | 1.05  ±0.09 | 1.02  ±0.16  (*thraa*)  1.04  ±0.21  (*thrab*) | 1.10  ±0.26  (*thraa*)  1.40  ±0.12  (*thrab*) | 2.75  ±0.14  (*thraa*)  5.20  ±0.13  (*thrab*) | 2.68  ±0.32  (*thraa*)  6.27  ±0.18  (*thrab*) |
| ***Thra2*** | | 1.04  ±0.20 | 2.08  ±0.55 | 3.07  ±0.73 | | | 1.02  ±0.12 | 3.26  ±0.20 | 4.59  ±0.57 | 1.01  ±0.08 | 1.63  ±0.17 | 0.82  ±0.06 | 1.02  ±0.13 | 1.05  ±0.10 | --- | --- | --- | --- |
| ***Thrb*** | | 1.04  ±0.19 | 0.92  ±0.32 | 1.35  ±0.43 | | | 1.00  ±0.04 | 1.06  ±0.16 | 1.43  ±0.33 | 1.01  ±0.09 | 2.82  ±0.43 | 5.51  ±0.54 | 1.03  ±0.17 | 3.34  ±0.23 | 1.02  ±0.16 | 1.15  ±0.10 | 0.70  ±0.03 | 0.60  ±0.07 |
| ***Ncor1*** | | 1.03  ±0.18 | 1.20  ±0.19 | 1.64  ±0.19 | | | 1.01  ±0.09 | 0.43  ±0.06 | 0.79  ±0.14 | 1.02  ±0.14 | 0.88  ±0.12 | 1.13  ±0.23 | 1.02  ±0.12 | 0.88  ±0.10 | 1.04  ±0.21 | 0.94  ±0.35 | 0.75  ±0.12 | 0.77  ±0.12 |
| ***Klf9*** | | 1.13  ±0.34 | 0.71  ±0.04 | 0.59  ±0.12 | | | 1.02  ±0.14 | 0.28  ±0.03 | 0.30  ±0.08 | 1.01  ±0.11 | 2.66  ±0.47 | 11.05  ±1.70 | 1.25  ±0.61 | 4.95  ±0.38 | 1.02  ±0.13 | 0.62  ±0.05 | 13.28  ±3.43 | 22.81  ±5.95 |
| ***myelin*** | | 1.04  ±0.21  (*MBP*) | 1.87  ±0.41  (*MBP*) | 16.1  ±6.36  (*MBP*) | | | 1.01  ±0.12  (*mobp*) | 13.74  ±3.03  (*mobp*) | 9.84  ±2.84  (*mobp*) | 1.26  ±0.50  (*mobp*) | 8.31  ±3.96  (*mobp*) | 0.60  ±0.36  (*mobp*) | 1.06  ±0.25  (*mobp*) | 50.70  ±13.0  (*mobp*) | 1.02  ±0.14  (*mbpa*) | 1.38  ±0.12  (*mbpa*) | 43.73  ±0.59  (*mbpa*) | 126.78  ±3.3  (*mbpa*) |
| ***Hr*** | | 1.05  ±0.25 | 1.00  ±0.17 | 1.64  ±0.24 | | | 1.02  ±0.13 | 0.84  ±0.04 | 1.07  ±0.31 | 1.00  ±0.05 | 0.80  ±0.13 | 0.75  ±0.25 | 1.03  ±0.16 | 3.70  ±0.25 | --- | --- | --- | --- |
|  | **No change** | | | |  |  |  |  |  |  |  |  |  |  |  |  |  |  |
|  | **Upregulated p < 0.05** | | | |  |  |  |  |  |  |  |  |  |  |  |  |  |  |
|  | **Downregulated p < 0.05** | | | |  |  |  |  |  |  |  |  |  |  |  |  |  |  |
