# Supplementary material for: Ontogenetic expression of thyroid hormone signaling genes: An in vitro and in vivo species comparison
Source: PLoS One. 2019 Sep 12;14(9):e0221230. doi: 10.1371/journal.pone.0221230 (PMC6742404; doi:10.1371/journal.pone.0221230)
Supplement: S3 Table — (DOCX) [file pone.0221230.s003.docx]

**Supplementary Table 3:** Primer sequences.

| **Gene Name** |  | **Rat Primer Sequence (5' - 3')** | **Human Primer Sequence (5' - 3')** | **Zebrafish Primer Sequence (5' - 3')** |
| --- | --- | --- | --- | --- |
| **Transporters** | | | | |
| *lat1* | F | TCATCATTCGGCCCTCCTCA | TCATCATCCGGCCTTCATCG | GTGGAGATCGGCAAAGGTGA |
|  | R | ACAGCCGTGAGTAGTAGCAC | TCACGCTGTAGCAGTTCACG | CAGCCCACTGTAAAGAGCCA |
| *lat2* | F | GGGCCTTCCTGCTGATTTTC | GGTCTTCAGCCTGTGGTCAG | ATGGACCGAGACAAAGAGGC |
|  | R | CACTTGGGTTTGTGTTGCCAG | TGGGCTTGTGTTGCCAGTAA | ATGTTACCGACAATAATGCCGCAC |
| *mct8* | F | ACATCAGTGACTCCATCCCT | CCACATCAGTGACTCCATCC | CTTCGGATGTCGGAAAACGG |
|  | R | AAGAGGCAGACAACGATAAGG | AAAGGCCCAGGAAAAGACAG | CCCAGAGTCGTGGCGAAG |
| *oatp1c1* | F | CAAGATGCAAATGTTCAGACTCA | AGCAGTATGGACAGTCATCCT | TGTTGATGTCACTCGGGACG |
|  | R | CAGCAAATCCCACACAAGTG | CTCCACACACACTGATTCTGA | TACAGACAAGCGGATCGACG |
| **Deiodinases** | | | | |
| *dio2* | F | TGGAGCGTTTCTCCTTGCC | AGCTTCTGGAGCGTTTCTCC | TGGATGCCTACAAACAGGTGA |
|  | R | ACGTTCAAAGGCTACCCCAT | ACCCCGTAAGCTATGTTGGC | GTCTTACCGCTGATGCTCCA |
| *dio3* | F | GCCCGTTGGTGCTCAATTTT | GCCCGCTGGTTCTCAATTTC | *Not used* |
|  | R | CTGTGGGATGACGTAGGGTG | CTTGCTGCAGTACCCTCGCT |  |
| *dio3a* | F | *Not used* | *Not used* | TCGCACCTGTATTCTCCGTG |
|  | R |  |  | CGAGCGTCCCGTATTCAGAC |
| *dio3b* | F | *Not used* | *Not used* | GCAGAGCGCATCCTGGATTA |
|  | R |  |  | CTCAGACGGGTCATGAAGGG |
| **TH Receptors and Corepressors** | | | | |
| *thra1* | F | TGCTGTTAATGTCAACAGACCGC | GCTGCTAATGTCAACAGACCGCT | *Not used* |
|  | R | CCCCGATCATGCGGAGGTCA | CCCCGATCATGCGGAGGTCA |  |
| *thraa* | F | *Not used* | *Not used* | CCAGCCGAAACCGAATCGT |
|  | R |  |  | CGATACCCTTCCAAACCGCT |
| *thrab* | F | *Not used* | *Not used* | CCCCGCGATCTCCCCAAAA |
|  | R |  |  | TGGGATGAGCTCAGTGTCCTG |
| *thra2* | F | GCTGCTGATGAAGGAGAGAGAA | GCTGCTGATGAAGGAGAGAGAA |  |
|  | R | GAGACTTCCCGCTTCACCAA | GAGACTTCCCGCTTCACCAA |  |
| *trb* | F | GCTGATAGAAGAGAACCGTGAG | GAACGACCAGAGTGTCTCAAG | GGGTCATTTCAGGCCACGTA |
|  | R | CCTCGGTGACTGTCTTGATG | GTCACCACACACTACACAGA | CAGATGAGAGACCCTCGAAGC |
| *ncor1* | F | GAATGGGCTCATGGAGGATCC | GAATGGGCTTATGGAGGACCC | TCGGGCTAATCGCCTCTTTT |
|  | R | GATTTCTGCCTCTGCGTTTC | GGTTTCTGCCTCTGCGTTTC | TGTTATCTGCTGATTCCGTCG |
| **TH-Responsive Genes** | | | | |
| *klf9* | F | CACCGAATCTGGGTCGAGTC | CACCGAATCTGGGTCGAGTC | GGGTGACTACGATGACGGAC |
|  | R | CCGTTCACCTGTATGCACTC | CCGTTCACCTGTATGCACTC | CTCCTCGCCGGTTAGTTTGT |
| *mobp* | F | ACCCATCTGCCCTCAGACTTA | *Not used* | *Not used* |
|  | R | GCATCTGTAGTTGTTACATCAGC |  |  |
| *mbp* | F | *Not used* | CAGAGCGTCCGACTATAAATCG | *Not used* |
|  | R |  | GGTGGGTTTTCAGCGTCTA |  |
| *mbpa* | F | *Not used* | *Not used* | ATCTCGCTCTCCACCCAAAC |
|  | R |  |  | GCGACTGGTGAGTCTGTAGG |
| *mbpb* | F | *Not used* | *Not used* | GGGCAGCATCTTGGAAAACG |
|  | R |  |  | TGTTCTGGTTGAGGTCTGCC |
| *hr* | F | CTGTCACCAGTGCGAACC | GAGCGAATACACATGGCCTT | *Not expressed* |
|  | R | CTGGATGCCTGATACTAACACA | CTTTCTCCTGGATCTTCCGTT |  |
| **Reference Genes** | | | | |
| *pgk1* | F | GAACCTCCGCTTTCATGTAGA  (rCNC) | *Not used* | *Not used* |
|  | R | AGCATCATTGACATAGACATCTCC (rCNC) |  |  |
| *ppia* | F | TTTGCAGACGCCGCTGT  (rCNC, rat cortex) | *Not used* | *Not used* |
|  | R | ATCAGCCGTGATGTCGAAG  (rCNC, rat cortex) |  |  |
| β-actin | F | CCTCTATGCCAACACAGT  (rNPCs, rat brain) | CAGGAAGTCCCTTGCCATCC | AAGCAGGAGTACGATGAGTC |
|  | R | AGCCACCAATCCACACAG  (rNPCs, rat brain) | ACCAAAAGCCTTCATACATCTCA | TGGAGTCCTCAGATGCATTG |
